# Relationship of HOMA-IR with chronic kidney disease in diabetic and non-diabetic Chinese populations: findings from the REACTION study

**DOI:** 10.3389/fendo.2026.1707947

**Published:** 2026-04-21

**Authors:** Yuheng Liao, Mijie Guan, Qijun Wan, Haiying Song, Haofei Hu

**Affiliations:** 1 Department of Nephrology, Shenzhen Second People’s Hospital, Shenzhen, Guangdong, China; 2 School of Medicine Shenzhen University, Shenzhen, China; 3 Department of Nephrology, The First Affiliated Hospital of Shenzhen University, Shenzhen, Guangdong, China; 4 Department of Vascular Access Center, The First Affiliated Hospital of Jinan University, Jinan University, Guangzhou, China

**Keywords:** Chronic kidney disease, cross-sectional study, diabetes mellitus, homeostasis model assessment of insulin resistance, insulin resistance, non-linear association

## Abstract

**Background:**

Chronic kidney disease (CKD) affects 8.2% of China’s population and is a major global health concern. While insulin resistance (IR) is linked to CKD, the relationship between insulin resistance (HOMA-IR) and CKD risk remains unclear, especially in diabetic and non-diabetic populations.

**Methods:**

This cross-sectional study analyzed data from 32,055 Chinese adults in the REACTION study. Logistic regression and generalized additive models assessed the association between HOMA-IR and CKD risk in diabetic (DM) and non-diabetic (Non-DM) populations, with nonlinear relationships explored using two-piecewise logistic regression.

**Results:**

The overall CKD prevalence was 16.09%(95% CI: 15.68%-16.49%). In the Non-DM group, HOMA-IR was positively associated with CKD risk (OR = 1.037, 95% CI: 1.010–1.066, P = 0.008), while no significant association was found in the DM group (OR = 0.991, 95% CI: 0.952–1.032, P = 0.667). Both groups showed an n-shaped relationship, with inflection points at HOMA-IR values of 2.581 (Non-DM) and 2.587 (DM). Below these thresholds, CKD risk increased with HOMA-IR; above them, risk decreased.

**Conclusion:**

Elevated HOMA-IR is independently associated with an increased risk of CKD in non-diabetic individuals, whereas this association is not significant in diabetic patients. These findings strongly highlight the clinical value of HOMA-IR as an early predictor of CKD risk, particularly in non-diabetic populations, emphasizing the importance of monitoring insulin resistance for early risk stratification and tailored management.

## Background

Chronic kidney disease (CKD) has become a worldwide public health problem. In the past 2 years, the age-specific mortality rate of CKD has almost doubled, and the age-specific prevalence of CKD has increased by 30% to 700 million people worldwide (1). CKD affects approximately 8.2% of the Chinese population (2). Furthermore, individuals diagnosed with CKD not only bear a substantial financial burden associated with renal therapy, but also demonstrate a heightened susceptibility to comorbid conditions, including cardiovascular diseases and diabetes, as well as elevated all-cause mortality (3, 4). Recognizing the modifiable behavioral risk factors of CKD is therefore essential for facilitating timely preventive and interventional strategies to mitigate serious health consequences (5).

Previous studies have demonstrated that insulin resistance (IR) plays a crucial role in the development and progression of chronic kidney disease (CKD). IR constitutes a prevalent and early-onset metabolic disturbance in CKD, emerging even at stages where the glomerular filtration rate (GFR) is still preserved within normal limits (6). As GFR progressively declines, IR becomes increasingly prevalent, reaching near-universal presence in end-stage renal disease (7, 8). However, whether IR is associated primarily with proteinuria or with reduced estimated GFR (eGFR) has not been well characterized — particularly in the context of diabetes — which limits our understanding of when and how IR affects the kidneys. Notably, IR has been demonstrated to be strongly linked to a range of conditions recognized as well-established contributors to CKD progression, encompassing diabetes mellitus(DM) (9), cardiovascular disease (CVD) (10), hypertension (11, 12),coronary heart disease (13), obesity (14, 15), dyslipidemia (16), and metabolic syndrome (17), which are well-established risk factors for CKD development. Consequently, the timely detection and therapeutic management of IR hold substantial potential in preventing or delaying the initiation and progression of CKD along with its attendant complications, thereby reinforcing the critical necessity of implementing systematic screening protocols and stringent control measures targeting IR within susceptible high-risk populations as well as individuals presenting with early-stage CKD.

The hyperinsulinemic-euglycemic clamp technique is broadly regarded as the reference standard method for the evaluation of IR (18). However, its invasive nature and impracticality render it unsuitable for large-scale epidemiological studies (19). In response to this limitation, the Homeostasis Model Assessment of Insulin Resistance (HOMA-IR) has been introduced by investigators as a practical alternative measurement. HOMA-IR has gained extensive application in both clinical settings and large-scale epidemiological investigations (20), and its validity and utility as a surrogate indicator for CKD evaluation have been well-corroborated by multiple studies (21–24).

An increasing body of evidence has demonstrated a significant association between HOMA-IR and CKD risk. Notably, a large-scale community-based prospective cohort study conducted in Korea over a 14-year follow-up period (25) revealed that HOMA-IR exhibited a statistically significant positive correlation with the incidence of CKD (HR = 1.12, 95%CI: 1.08-1.15, p=0.001), while also displaying considerable predictive capacity for CKD (AUC: 0.767, 95%CI: 0.742-0.791, p=0.015). In a retrospective cross-sectional study performed in China, following adjustment for potential confounding variables, individuals in the fourth quartile of HOMA-IR demonstrated a statistically significant positive association with the likelihood of CKD development relative to those in the first quartile (males: OR, 2.30; 95% confidence interval 1.50–3.51; P < 0.001; females: OR, 2.20; 95% confidence interval 1.35–3.58; P = 0.002) (26).

However, certain investigators have argued that HOMA-IR does not function as an independent predictor of CKD (18, 24). Evidence from a multicenter prospective observational cohort study (24) indicated that, upon adjustment for covariates, HOMA-IR failed to demonstrate a statistically significant association with CKD progression (HR = 1.01, 95%CI: 0.90–1.14). A multi-center cross-sectional study (27) in China reported that HOMA-IR was significantly associated with the presence of CKD in the general population (OR = 1.549,95%CI:1.079-2.223, P = 0.018). However, this association lost significance when patients with metabolic syndrome were excluded (OR = 1.314, 95%CI: 0.803-2.151). In addition, another cross-sectional study in China involving 5061 participants revealed different results of HOMA-IR and CKD by gender (28). Multivariate logistic regression showed that HOMA-IR was significantly associated with the prevalence of CKD in men, but not in women (male = 1.21; 95%CI:1.14-1.28, p ≤ 0.001; female = 1.01; 95%CI:0.99-1.02, p = 0.38).These conflicting results highlight the ongoing controversy surrounding the HOMA-IR-CKD relationship.

HOMA-IR represents a extensively utilized indicator for evaluating the risk and advancement of type 2 diabetes, and has been well-documented to maintain a strong correlation with the likelihood of CKD development (29). However, only a limited number of studies have examined the effect of diabetes status on the relationship between HOMA-IR and CKD, and the findings have been inconsistent. A Korean study conducted in a non-diabetic population (23) demonstrated that elevated HOMA-IR was an independent risk factor for CKD (OR = 1.49, 95% CI: 1.12–1.98; p = 0.01). In contrast, another Korean study reported contradictory findings across diabetic and non-diabetic subgroups: while insulin resistance was positively associated with CKD in Korean adults both with and without type 2 diabetes, HOMA-IR was not independently associated with CKD in the non-diabetic subgroup after adjustment for relevant covariates, with the exception of age (p = 0.163) (30). Similarly, a Chinese study (31) found that CKD risk increased significantly across ascending quartiles of HOMA-IR among individuals with diabetes after adjustment for confounding factors (OR: 1.37, 95% CI: 1.07–1.74); however, this association did not reach statistical significance among those without diabetes (OR: 1.03, 95% CI: 0.89–1.20).

In this regard, we hypothesized that the relationship between HOMA-IR and CKD risk may differ between diabetic and non-diabetic populations, and that the utility of HOMA-IR as a tool for identifying and evaluating CKD warrants further investigation. Therefore, the primary aim of this study was to examine the association between HOMA-IR and CKD risk in both diabetic and non-diabetic Chinese adults, and to determine whether this association differs according to diabetes status.

## Methods

### Study design

The present investigation employed a cross-sectional design to examine the relationship between HOMA-IR and CKD among individuals with and without diabetes. HOMA-IR served as the primary independent variable of interest, while CKD was designated as the dependent variable, operationalized as a binary outcome (1 = CKD, 0 = non-CKD).

### Data source

The data utilized in this derived analysis were sourced from the REACTION (Risk Evaluation of Cancers in Chinese Diabetic Individuals: A Longitudinal) study, a large-scale, multicenter, community-based prospective observational cohort investigation. The primary objective of the original REACTION study was to examine the epidemiological characteristics of abnormal glucose metabolism and its relationship with the susceptibility to cancer and other chronic conditions within the Chinese population (32). The raw dataset was made publicly available by Ye et al. under the Creative Commons Attribution License and is accessible through the PLOS ONE data repository at https://doi.org/10.1371/journal.pone.0214776.s003.

### Statement of ethics

The primary study was carried out in full compliance with the principles outlined in the Declaration of Helsinki and received ethical clearance from the Committee on Human Research at Rui-Jin Hospital, affiliated with Shanghai Jiao Tong University School of Medicine. Written informed consent was duly obtained from all enrolled participants. Of particular note, given that the current study constitutes a secondary analysis of a completely anonymized, publicly accessible dataset, the obligation for additional ethical approval and informed consent specific to this derived analysis was formally exempted by the Institutional Review Board of Shenzhen Second People’s Hospital.

### Study population

This study utilized data from the Risk Evaluation of Cancers in Chinese Diabetic Individuals: A Longitudinal (REACTION) study, which comprised 33,850 participants recruited from eight regional centers across China (Shanghai, Guangzhou, Wuhan, Dalian, Zhengzhou, Lanzhou, Luzhou, and Guangxi). The REACTION study enrolled community-dwelling adults who were representative of the general Chinese population. The current analysis was based on this well-established population-based cohort database.

The original study applied the following exclusion criteria: (1) individuals diagnosed with primary kidney disease; (2) participants receiving daily antihypertensive therapy with ACEI or ARB medications; (3) subjects reporting an implausible self-reported sleep duration (<4 hours or >12 hours). Following the application of these criteria, a total of 33,850 individuals were ultimately retained for inclusion in the final analysis.

**Figure 1:**
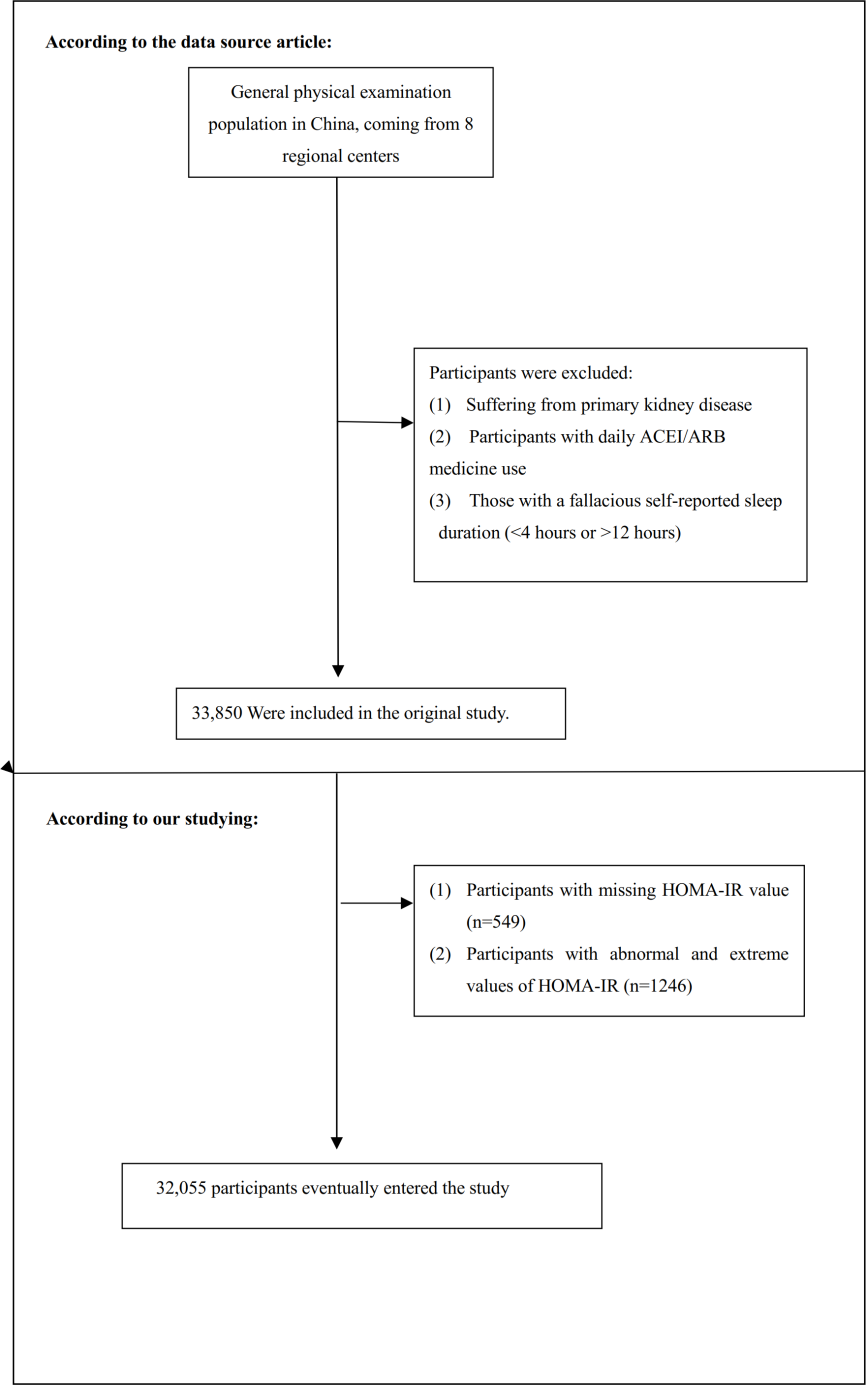
Flow chart of study participant selection from the REACTION study. This flow chart illustrates the stepwise selection process of the study cohort. Of the initial 33,850 participants recruited from the general Chinese population across 8 regional centers, exclusions were made based on predefined criteria, including missing data and extreme HOMA-IR or CKD values. The final analysis included 32,055 participants. Abbreviations: ACEI/ARB, Angiotensin-Converting Enzyme Inhibitor/Angiotensin II Receptor Blocker; HOMA-IR, Homeostasis Model Assessment of Insulin Resistance.

In this current study, following the original research, we further excluded individuals with missing HOMA-IR values (n=549) and those with HOMA-IR values beyond three standard deviations from the mean (n=1246) (33), and a total of 32,055 participants were ultimately enrolled in the study. **Figure 1** illustrates the process of participant selection.

In this cohort, there was a disproportionate number of non-diabetic (n=24,891) and diabetic (n=7,164) participants. This imbalance is attributable to the community-based epidemiological nature of the REACTION study. Because the original study recruited community-dwelling adults via natural population screening rather than exclusively enrolling patients from specialized diabetes clinics, the composition naturally reflects the real-world prevalence of diabetes among the general Chinese adult population. Despite this disproportion, the absolute sample size of the diabetic group remains robust enough to ensure sufficient statistical power for all stratified analyses.

### Variables

#### Data collection and measurements

Fundamental demographic characteristics, detailed medical history, and relevant lifestyle-related factors — encompassing physical activity levels, smoking status, and alcohol consumption patterns — were systematically collected through the administration of a standardized structured questionnaire. Both smoking habits and alcohol consumption behaviors were independently classified into three distinct categories, respectively defined as regular users, occasional users, and never users. Among all variables examined in this study, information regarding smoking status and alcohol consumption was missing for 281 and 263 participants, respectively. To maintain statistical power and prevent potential selection bias, participants with missing data were retained in the analyses and categorized as “Not recorded” rather than excluded from the study population (34).

Standardized baseline assessments were carried out by trained healthcare professionals. Anthropometric parameters encompassed height (recorded to the nearest 0.1 cm using a stadiometer), body weight (recorded to the nearest 0.1 kg using calibrated electronic scales), and waist circumference (obtained at the horizontal midpoint between the anterior superior iliac spine and the inferior margin of the 12th rib). Blood pressure was recorded on three consecutive occasions at 1-minute intervals following 5 minutes of rest in a seated position, with the arithmetic mean of the three readings subsequently employed for statistical analysis. Hypertension was operationally defined as a systolic blood pressure (SBP) ≥130 mmHg, a diastolic blood pressure (DBP) ≥80 mmHg, or a documented prior diagnosis of hypertension.

Morning venous blood specimens were obtained from all participants following a mandatory 8-hour overnight fasting period. Participants without diabetes were subsequently administered a 75-g oral glucose tolerance test, whereas those with a confirmed diagnosis of diabetes underwent a 100-g steamed bread tolerance test. Peripheral venous blood specimens were collected at baseline and at 120 minutes following the oral glucose challenge. The biochemical index included triglycerides (TG), total cholesterol (TC), high-density lipoprotein cholesterol (HDL-c), low-density lipoprotein cholesterol (LDL-c), serum creatinine (Scr), urea nitrogen (BUN), alanine aminotransferase (ALT), aspartate aminotransferase (AST), γ-Glutamyl transpeptidase (GGT), fasting plasma glucose (FPG), glycosylated hemoglobin (HbA1c), and postprandial blood glucose (PBG), which were measured using the glucose oxidase-peroxidase method. Diabetes mellitus (DM) was defined by any of the following criteria: FPG ≥7.0 mmol/L, PBG ≥11.1 mmol/L, or documented history of diabetes (35).

### Calculation and assessment of HOMA-IR

The HOMA-IR index was computed through a sequential two-step calculation procedure. In the first step, fasting plasma insulin (FPI) was quantified in microunits per milliliter (μU/mL), while fasting plasma glucose (FPG) was simultaneously determined in milligrams per deciliter (mg/dL). Second, the HOMA-IR index was calculated according to the following formula: HOMA-IR = [FPI (μU/mL) × FPG (mmol/L)]/22.5 (36).

### Definition of chronic kidney disease

CKD was defined as albuminuria (urinary albumin-to-creatinine ratio [UACR] ≥30 mg/g) or reduced estimated glomerular filtration rate (eGFR <60 mL/min/1.73 m²) (2). Morning urine samples were collected for UACR measurement. The eGFR was calculated using the Modification of Diet in Renal Disease equation: eGFR (ml/min*1.73 m2) =186*Scr (mg/dl)-1.154*Age (years)-0.203(female*0.742) (37).

### Assessment of covariates

The selection of variables in our research was guided by primary investigation, clinical expertise, and previous studies on the risk of CKD (38, 39). The analyzed covariates comprised two categories: (1) Continuous variables: age, BMI, WC, HC, HDL-c, LDL-c, TG, TC, AST, ALT, GGT, eGFR, UACR. (2) Categorical variables encompassed demographic characteristics (sex), lifestyle-related factors (occupational status, smoking behavior, and alcohol consumption habits), as well as history of chronic conditions (comprising hypertension and malignancy).

### Statistical analysis

Participants were initially stratified into two subgroups — a diabetic group and a non-diabetic group — based on the presence or absence of a confirmed diabetes diagnosis. Subsequently, all continuous variables were assessed for distributional normality through the Kolmogorov-Smirnov test in conjunction with visual examination of frequency histograms. Continuous variables conforming to a normal distribution are reported as means ± standard deviations (SD), and between-group comparisons were carried out using the Student’s t-test or one-way analysis of variance (ANOVA), as appropriate. Continuous variables demonstrating a non-normal distribution (including HOMA-IR, triglycerides, AST, and ALT) are summarized as medians accompanied by interquartile ranges (IQR), with inter-group differences evaluated by the non-parametric Mann-Whitney U test or the Kruskal-Wallis test, accordingly. Categorical variables are expressed as absolute frequencies and corresponding percentages (n, %), and between-group comparisons were conducted by means of the Pearson’s Chi-square test (33).

Potential multicollinearity among covariates was assessed by computing variance inflation factors (VIF) (40), derived according to the formula 1/(1-R²), in which R² was obtained from a series of sequential linear regression models with each individual variable designated as the dependent variable regressed against the remaining covariates. Any variable yielding a VIF exceeding 5 was subsequently removed from the multivariable regression analysis on the grounds of substantial collinearity (**Supplementary Table 1**).

The association between HOMA-IR and CKD risk in people with and without diabetes was evaluated using three logistic regression models following STROBE guidelines (41). In order to delineate the independent association between HOMA-IR and the risk of CKD while accounting for the influence of potential confounding variables, a series of multivariable logistic regression models were subsequently established. Variables that demonstrated significant baseline differences or clinical relevance were incorporated into the fully adjusted model (Model III) simultaneously. Specifically, Model I without adjusting for covariates, Model II only adjusting for sociodemographic variables, including age, gender, and BMI; Model III was simultaneously adjusted for age, sex, BMI, ALT, AST, GGT, HDL-c, TG, smoking, drinking, physical exercise, hypertension, and history of malignancy. This simultaneous adjustment strategy ensures the neutralization of demographic and biochemical confounders, thereby evaluating the independent, basal pathogenic effect of HOMA-IR. Covariate selection was guided by previous literature (23, 25, 42, 43), and collinearity assessment. Owing to its confirmed collinear relationship with other covariates, total cholesterol was subsequently omitted from the multivariable regression analysis.

To explore potential nonlinear dose-response relationships between HOMA-IR and CKD, generalized additive models (GAM) incorporating penalized spline smoothing functions were utilized. Upon confirmation of a nonlinear pattern, the threshold inflection point was subsequently identified through the application of a recursive algorithm, followed by segmented two-piece logistic regression analyses performed on either side of the identified threshold. Model selection between linear and nonlinear specifications was carried out based on the log-likelihood ratio test (44).

We conducted stratified binary logistic regression model across key demographic and clinical parameters. Age was categorized into five groups (<=40, >40 to<=50, >50 to <=60, >60 to <=70, >70 years), while clinical parameters were dichotomized using established thresholds (45): SBP (<130, ≥130 mmHg), DBP (<80, ≥80 mmHg). Within each stratified subgroup, all covariates were retained as adjustment variables with the sole exception of the variable employed for stratification. The presence of interaction effects was examined by means of likelihood ratio tests, through direct comparison of models incorporating interaction terms against corresponding models without such terms (46).

Given the absence of universally accepted clinical cut-off values for HOMA-IR, we adopted a dual-approach statistical strategy to comprehensively evaluate its association with CKD. First, to avoid arbitrary thresholding and facilitate clinical interpretability, HOMA-IR was categorized into three subgroups (tertiles, Q1-Q3) based on the data distribution of the cohort. This data-driven categorization allowed for the evaluation of dose-response relationships by calculating the P value for trend. Second, and more importantly, to prevent the loss of information and maximize statistical power, HOMA-IR was fundamentally analyzed as a continuous variable in all multivariable logistic regression models (calculating the risk per 1-unit increment) and Generalized Additive Models. All methods and results are reported in accordance with the STROBE statement (41).

Statistical analyses were conducted using R (The R Foundation; http://www.R-project.org) and EmpowerStats (X&Y Solutions, Inc; http://www.empowerstats.com). Two-sided tests with P<0.05 defined statistical significance.

## Results

### Characteristics of participants

A total of 32055 participants were included (67.17% were female), with an average age of (57.6 ± 9.3) years. **Table 1** summarizes the demographic and clinical characteristics of the study population. The mean HOMA-IR was 1.87 (1.29-2.72), the mean BMI was 24.64 ± 3.69 kg/m², the mean eGFR was 94.16 ± 19.02 ml/min/1.73m^2^, and the median UACR was 9.93 (5.75,19.87)mg/g. The prevalence of CKD was 16.09% (95% CI: 15.68%-16.49%). We first divided all participants into DM and Non-DM groups. According to HOMA-IR, participants in each group were further divided into three subgroups (<1.063, ≥1.063 to <3.365, and ≥3.665). In the DM group, compared with the T1 (<1.063) group, the T3 (≥3.665) group had higher age, male, female, MAP, AST, FPG, 2hPG, HbA1c, eGFR, UACR, and a higher proportion of male and CKD. In addition, no statistical significance was observed in BMI, HC, HDL-c, AST, FPG, Fasting insulin, and CKD. In contrast, T3 showed lower levels of BMI, WC, HC, Scr, LDL-c, HDL-c, TG, TC, GGT, ALT, and fasting insulin, as well as lower proportions of exercise status, hypertension, tumors, and smoking/drinking habits. However, in the non-DM group, the T3 group had higher levels of age,2hPG, HbA1c, eGFR, and UACR than the T1 group, as well as higher proportions of obesity, CKD, hypertension, cancer, and smoking/drinking status. In contrast, T3 had lower levels of male and female, MAP, LDL-c, TC, Scr and a lower proportion of exercise status (**Table 1**). In addition, no statistical significance was observed for HC, BMI, Fasting insulin, and Obesity.

**Table 1:** Baseline characteristics according to HOMA tertiles stratified by diabetes status.

Characteristics	Non-DM				DM				P-value
	HOMA-IR tertile			P-value	HOMA-IR tertile			P-value	
	T1(<1.063)	T2(≥1.063 T2 <3.365)	T3(≥3.665)		T1(<1.063)	T2(≥1.063 T2 <3.365)	T3(≥3.665)		
N	8619	8314	7958		2065	2372	2727		
Age, years	57.2 ± 10.2	55.4 ± 8.0	57.6 ± 8.7	<0.001	61.3 ± 10.6	59.2 ± 8.6	61.8 ± 8.4	<0.001	<0.001
Gender,n%									
Male	2987 (34.7)	2241 (27.0)	2350 (29.5)	<0.001	979 (47.4)	929 (39.2)	1038 (38.1)	<0.001	<0.001
Female	5632 (65.3)	6073 (73.0)	5608 (70.5)		1086 (52.6)	1443 (60.8)	1689 (61.9)		
BMI, kg/m²	24.3 ± 3.4	24.3 ± 3.6	24.5 ± 3.8	0.066	25.6 ± 3.5	25.7 ± 3.8	25.5 ± 3.9	0.194	<0.001
MAP, mmHg	94.4 ± 12.6	92.6 ± 12.4	94.1 ± 12.9	<0.001	99.3 ± 12.3	96.6 ± 12.5	98.1 ± 12.9	<0.001	<0.001
WC, cm	86.1 ± 9.7	84.5 ± 10.2	85.3 ± 9.8	<0.001	90.2 ± 9.4	89.2 ± 10.6	89.0 ± 9.5	<0.001	<0.001
HC, cm	96.5 ± 7.7	96.7 ± 7.5	96.5 ± 7.9	0.154	98.7 ± 8.2	98.7 ± 8.2	98.6 ± 8.3	0.989	<0.001
Scr, μmol/L	69.1 ± 14.9	68.0 ± 14.4	65.3 ± 15.2	<0.001	73.5 ± 23.9	71.2 ± 16.9	69.0 ± 18.9	<0.001	<0.001
HDL-c, mmol/L	1.4 ± 0.3	1.3 ± 0.3	1.3 ± 0.3	<0.001	1.3 ± 0.3	1.2 ± 0.3	1.2 ± 0.3	0.053	<0.001
LDL-c, mmol/L	3.1 ± 0.9	3.0 ± 0.9	2.8 ± 0.9	<0.001	3.2 ± 0.9	3.0 ± 0.9	2.9 ± 0.9	<0.001	0.307
TC, mmol/L	5.2 ± 1.1	5.0 ± 1.1	4.9 ± 1.2	<0.001	5.3 ± 1.2	5.0 ± 1.2	4.9 ± 1.2	<0.001	0.189
TG, mmol/L	1.5 ± 0.8	1.6 ± 0.8	1.5 ± 0.8	<0.001	1.8 ± 1.0	1.9 ± 1.0	1.8 ± 1.0	<0.001	<0.001
ALT, U/L	17.4 ± 12.6	17.5 ± 12.2	16.3 ± 11.9	<0.001	22.3 ± 18.5	20.9 ± 17.7	19.3 ± 17.0	<0.001	<0.001
AST, U/L	21.5 ± 10.4	22.1 ± 9.6	22.1 ± 10.3	<0.001	23.7 ± 14.2	23.7 ± 17.6	23.6 ± 18.7	0.875	
GGT, U/L	27.1 ± 25.4	26.8 ± 26.6	26.9 ± 30.1	<0.001	38.1 ± 52.7	37.6 ± 54.1	37.6 ± 61.4	<0.001	<0.001
FPG, mmol/L	5.4 ± 0.5	5.3 ± 0.6	5.5 ± 0.5	<0.001	7.9 ± 2.6	8.0 ± 2.7	7.9 ± 2.5	0.122	
2hPG, mmol/L	6.7 ± 1.7	7.0 ± 1.7	7.2 ± 1.7	<0.001	13.7 ± 4.5	13.7 ± 4.4	14.2 ± 4.7	<0.001	<0.001
HbA1c, %	5.7 ± 0.4	5.8 ± 0.4	5.8 ± 0.4	<0.001	7.1 ± 1.5	7.4 ± 1.6	7.2 ± 1.6	<0.001	<0.001
Fasting insulin, μU/mL	8.0 ± 4.3	7.9 ± 3.9	7.9 ± 4.0	0.084	11.0 ± 12.2	10.7 ± 10.6	10.5 ± 12.4	0.107	
eGFR, mL/min/1.73m²	93.2 ± 17.4	92.9 ± 16.7	98.2 ± 20.8	<0.001	90.2 ± 18.3	91.6 ± 20.4	94.5 ± 22.4	<0.001	<0.001
UACR, mg/g	24.0 ± 220.4	36.5 ± 638.7	26.9 ± 225.9	<0.001	47.4 ± 374.8	39.8 ± 258.0	53.0 ± 319.8	<0.001	<0.001
Smoking habits,n%									
Never smoker	1130 (13.1)	958 (11.5)	876 (11.0)	<0.001	326 (15.8)	333 (14.0)	313 (11.5)	<0.001	<0.001
Sometimes smoker	191 (2.2)	213 (2.6)	189 (2.4)		67 (3.2)	85 (3.6)	73 (2.7)		
Regular smoker	7259 (84.2)	7090 (85.3)	6773 (85.1)		1662 (80.5)	1933 (81.5)	2315 (84.9)		
Not record	39 (0.5)	53 (0.6)	120 (1.5)		10 (0.5)	21 (0.9)	26 (1.0)		
Drinking habits,n%									
Never drinker	648 (7.5)	438 (5.3)	516 (6.5)	<0.001	212 (10.3)	180 (7.6)	183 (6.7)	<0.001	<0.001
Sometimes drinker	1533 (17.8)	1745 (21.0)	1504 (18.9)		347 (16.8)	387 (16.3)	468 (17.2)		
Regular drinker	6397 (74.2)	6079 (73.1)	5830 (73.3)		1497 (72.5)	1785 (75.3)	2049 (75.1)		
Not record	41 (0.5)	52 (0.6)	108 (1.4)		9 (0.4)	20 (0.8)	27 (1.0)		
Exercise status,n%									
No	5838 (67.7)	6387 (76.8)	6939 (87.2)	<0.001	1509 (73.1)	1931 (81.4)	2541 (93.2)	<0.001	<0.001
Yes	2750 (31.9)	1896 (22.8)	1001 (12.6)		549 (26.6)	432 (18.2)	174 (6.4)		
Not record	31 (0.4)	31 (0.4)	18 (0.2)		7 (0.3)	9 (0.4)	12 (0.4)		
Hypertension,n%	5022 (58.3)	4340 (52.2)	4608 (57.9)	<0.001	1635 (79.2)	1671 (70.4)	2057 (75.4)	<0.001	<0.001
Obesity,n%	1155 (13.4)	1067 (12.8)	1076 (13.5)	0.381	448 (21.7)	554 (23.4)	552 (20.2)	0.027	<0.001
CKD,n%	1006 (11.7)	1064 (12.8)	1292 (16.2)	<0.001	501 (24.3)	572 (24.1)	723 (26.5)	0.087	<0.001
Tumor,n%	268 (3.1)	190 (2.3)	222 (2.8)	0.004	83 (4.0)	68 (2.9)	92 (3.4)	0.106	0.003

Data are presented as mean ± standard deviation (SD) for normally distributed continuous variables, median (interquartile range [IQR]) for skewed continuous variables, and number (percentage) for categorical variables.

### The prevalence rate of CKD

In the study population, 5158 participants (16.09%(95% CI: 15.68%-16.49%)) were diagnosed as CKD. In the DM group, the prevalence of CKD in the three HOMA-IR groups was 24.26% (22.41-26.11%), 24.11%(22.39-25.84%) and 26.51% (24.85-28.17%), respectively. However, in the non-DM group, the prevalence of CKD in the three HOMA-IR groups was 11.67% (10.99-12.35%), 12.80% (12.08-13.52%), and 16.24% (15.42-17.05%), respectively (**Table 2**).

**Table 2:** Prevalence of CKD in patients with and without diabetes.

HOMA-IR	Participants(n)	CKD (n)	Prevalence rate (95% CI)(%)
Total	32055	5158	16.09 (15.68-16.49)
Non-DM			
T1	8619	1006	11.67 (10.99-12.35)
T2	8314	1064	12.80 (12.08-13.52)
T3	7958	1292	16.24 (15.42-17.05)
Total	24891	3362	13.51 (13.08-13.93)
P for trend			<0.0001
DM			
T1	2065	501	24.26 (22.41-26.11)
T2	2372	572	24.11 (22.39-25.84)
T3	2727	723	26.51 (24.85-28.17)
Total	7164	1796	25.07 (24.06-26.07)
P for trend			<0.0001

CI, confidence; Ref, reference; HOMA-IR, homeostasis model assessment of insulin resistance; CKD, chronic kidney disease; DM, Diabetes mellitus, Non-DM, No diabetes mellitus.

To further elucidate the clinical stages of renal damage, we stratified the population based on UACR into three distinct categories: normoalbuminuria (< 30 mg/g), microalbuminuria (30–300 mg/g), and macroalbuminuria (> 300 mg/g) (47). Our analysis revealed that higher HOMA-IR tertiles were significantly associated with the progression from the <30 mg/g stage to the 30–300 mg/g stage (the reversible phase of CKD), particularly in the non-diabetic cohort (P < 0.001). However, in the diabetic cohort, the proportional increase of participants progressing from the 30–300 mg/g stage to the irreversible >300 mg/g stage was blunted, suggesting an uncoupling of HOMA-IR severity from progressive albuminuria in this specific subpopulation (**Supplementary Figure 1**).

**Figure 2:**
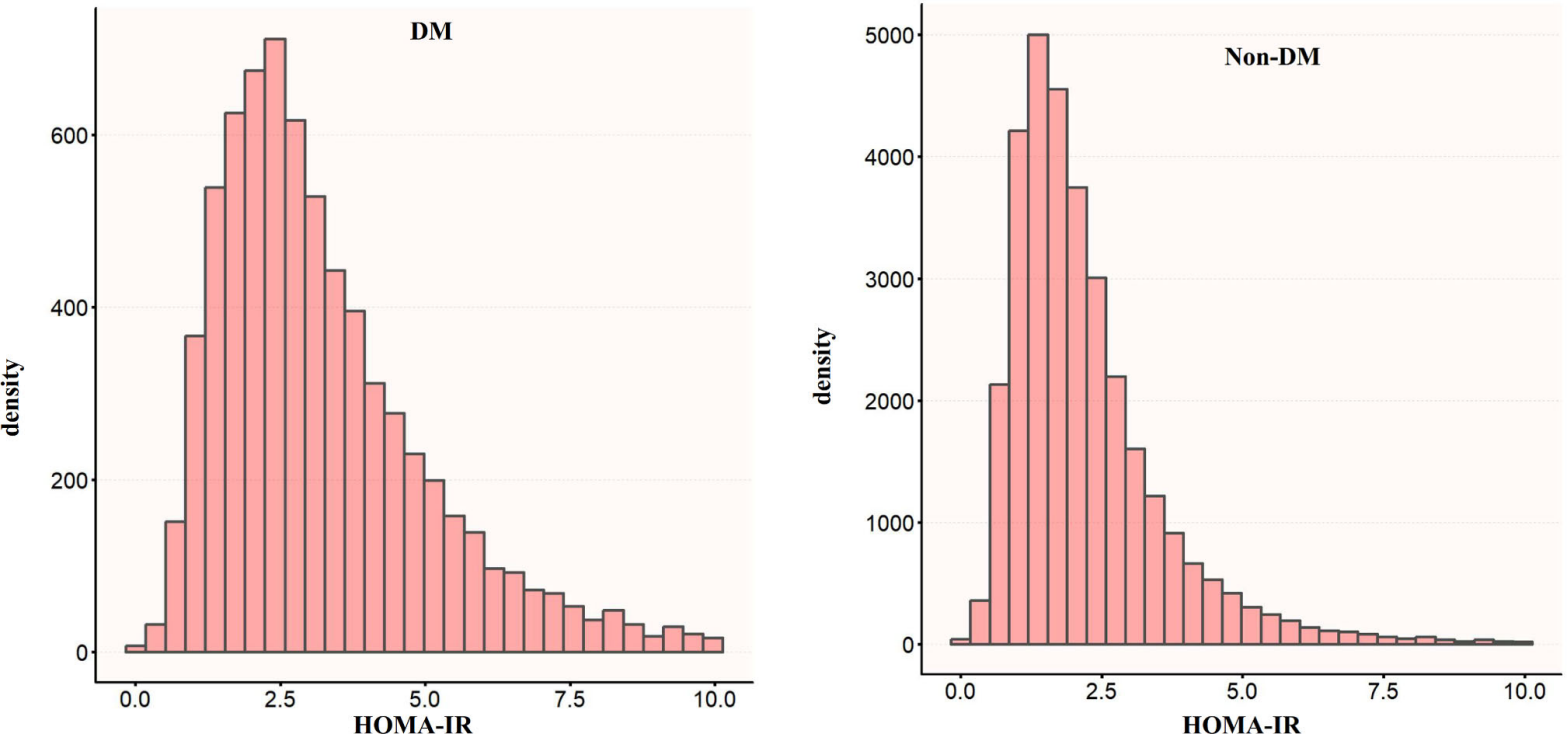
Distribution density of HOMA-IR levels stratified by diabetes status. The histograms display the right-skewed frequency distribution of HOMA-IR values in participants with diabetes (DM, red/top) and without diabetes (Non-DM, blue/bottom). The x-axis represents HOMA-IR values, and the y-axis represents the population density. DM, Diabetes Mellitus; Non-DM, Non-Diabetes Mellitus.

**Figure 3:**
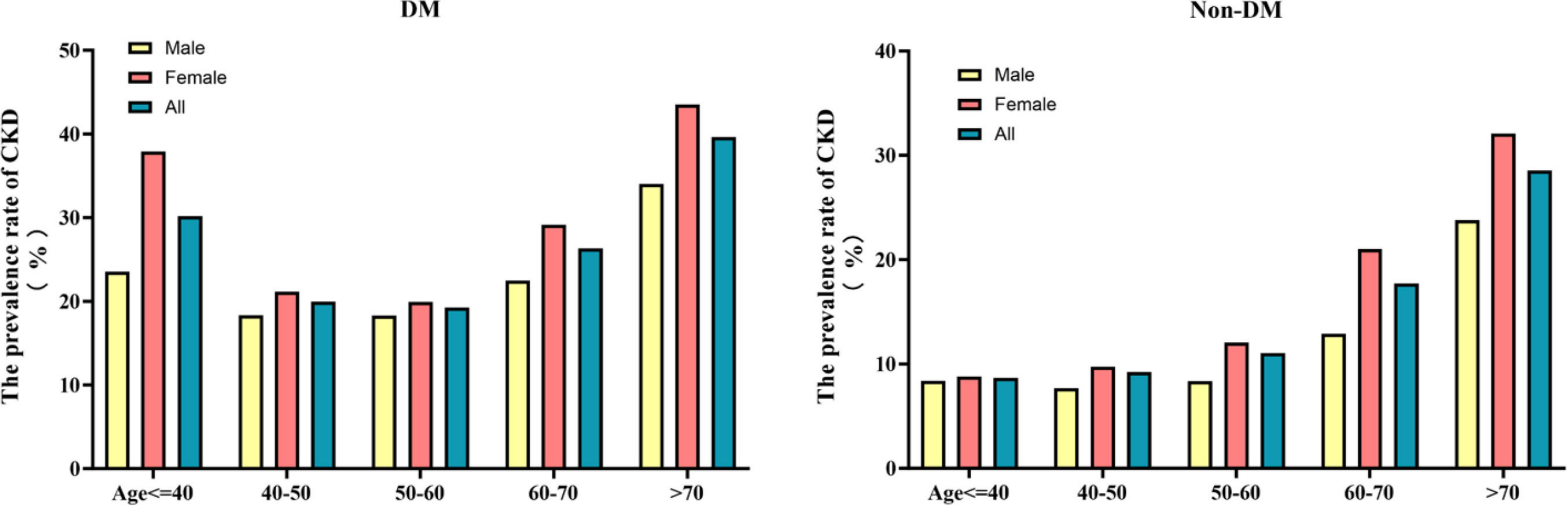
Age- and sex-specific prevalence of CKD in populations with and without diabetes. The bar charts demonstrate the prevalence of CKD across five age strata (≤40, 40-50, 50-60, 60-70, >70 years) and by sex (Male, Female, All). Data are presented separately for diabetic (DM) and non-diabetic (Non-DM) cohorts. In both cohorts, CKD prevalence increases progressively with age and exhibits sex-based disparities. CKD, Chronic Kidney Disease.

**Figure 2** shows the distribution of HOMA-IR levels in patients with or without diabetes. The level of HOMA-IR in DM group was right-skewed (range: 0.023 to 9.961, mean: 2.306). HOMA-IR in the non-DM group showed a right-skewed distribution (range: 1.340 to 4.794, mean: 2.175). When age groups were stratified by 10 intervals, the prevalence of CKD increased with age regardless of whether they had diabetes or not. The prevalence of CKD in women was generally higher than that in men, especially in the elderly (>60 years old) (**Figure 3**).

**Figure 4:**
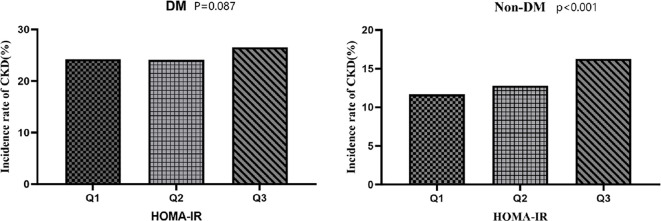
Prevalence of CKD across HOMA-IR tertiles in different diabetes statuses. The bars represent the prevalence rate of CKD categorized by HOMA-IR tertiles (Q1: lowest, Q2: middle, Q3: highest). In the Non-DM group, CKD prevalence increases significantly across tertiles (P for trend < 0.001). In the DM group, although an upward visual trend is present, the difference did not reach statistical significance (P = 0.087). Statistical significance was determined using the Cochran-Armitage test for trend.

The study population was divided into two groups according to whether diabetes was present, and then all participants were divided into three groups by HOMA-IR tertiles. **Figure 4** shows that the prevalence of CKD increased with the growth of HOMA-IR group in the Non-DM group, and the trend test was statistically significant (P < 0.001). However, in the DM group, although increased HOMA-IR was associated with increased prevalence of CKD, it did not reach statistical significance (p=0.087), possibly due to the high baseline risk of diabetes itself.

### Data visualization of HOMA-IR of all participants in different disease combinations

**Figure 5:**
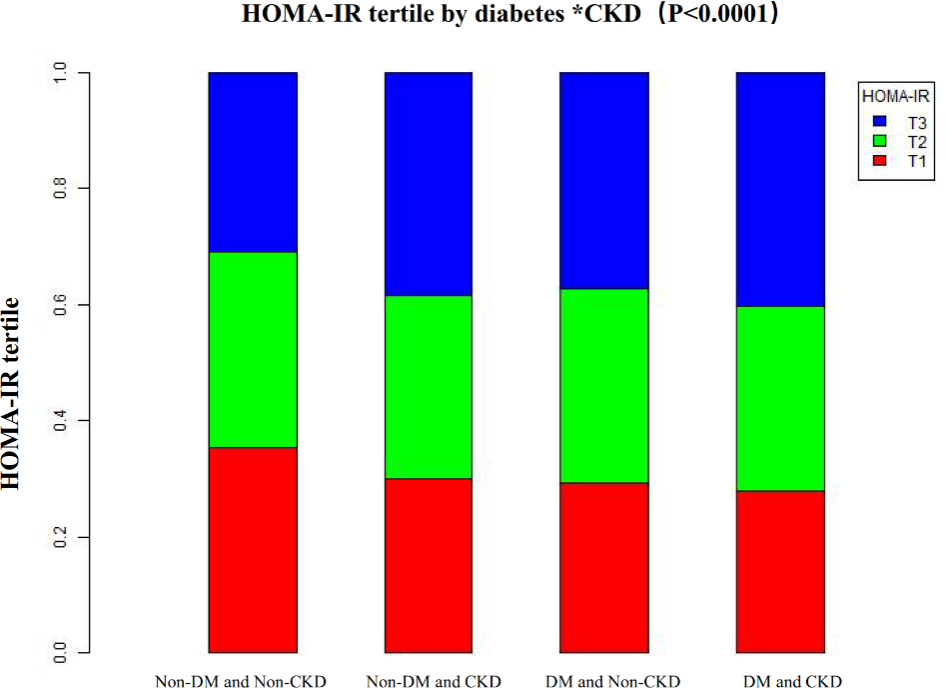
Distribution of high HOMA-IR (Tertile 3) across different disease combinations. The bar chart visualizes the proportion of individuals in the highest HOMA-IR tertile (T3) among four distinct subpopulations: Non-DM/Non-CKD, Non-DM/CKD, DM/Non-CKD, and DM/CKD. The highest prevalence of severe insulin resistance is observed in individuals suffering from both diabetes and CKD simultaneously.

Differences in tertiles of HOMA-IR in people with different disease combinations (DM and CKD). The proportion of high HOMA-IR group (T3) was higher in people with both CKD and DM than in other groups, such as non-CKD and non-DM group, CKD and non-DM group, and non-CKD and DM group. However, the proportion of high quantiles of HOMA-IR was approximately the same in patients with diabetes without CKD and those with CKD without diabetes. This suggests that both diabetes and CKD are strongly associated with increased insulin resistance and that insulin resistance is most severe when both are present (**Figure 5**).

### The results of univariate analyses using a binary logistic regression model

Univariate analysis showed that age, BMI, WC, MAP, Scr, TG, AST, GGT, FPG, 2hPG, HbA1c, fasting insulin, UACR and the proportion of regular smoking, regular drinking, hypertension and obesity were positively correlated with the risk of CKD regardless of the presence or absence of diabetes, However, the proportion of exercise status and eGFR level were negatively correlated with CKD. Meanwhile, HC was positively associated with CKD risk only in the non-DM group. HDL-c and LDL-c were negatively correlated with CKD in the non-DM group, but not in the DM group. ALT levels and tumor proportion were not significantly associated with CKD risk regardless of the presence or absence of diabetes (**Table 3**).

**Table 3:** The results of univariate analysis in patients with and without diabetes.

Variables	Non-DM	P value	DM	P-value
Female	1.313 (1.209-1.426)	<0.00001	1.285 (1.151-1.434)	<0.00001
Age, years	1.048 (1.044-1.052)	<0.00001	1.038 (1.032-1.044)	<0.00001
BMI, kg/m²	1.026 (1.016-1.035)	<0.00001	1.021 (1.006-1.035)	0.00411
MAP, mmHg	1.025 (1.022-1.027)	<0.00001	1.018 (1.014-1.023)	<0.00001
WC, cm	1.013 (1.010-1.017)	<0.00001	1.009 (1.004-1.014)	0.00125
HC, cm	1.014 (1.010-1.019)	<0.00001	1.006 (1.000-1.013)	0.05913
Scr, μmol/L	1.027 (1.025-1.030)	<0.00001	1.032 (1.028-1.035)	<0.00001
HDL-c, mmol/L	0.705 (0.631-0.787)	<0.00001	0.922 (0.777-1.095)	0.35479
LDL-c, mmol/L	0.913 (0.876-0.952)	0.00002	1.011 (0.956-1.070)	0.69776
TC, mmol/L	0.960 (0.928-0.992)	0.01424	1.048 (1.002-1.096)	0.04086
TG, mmol/L	1.225 (1.177-1.276)	<0.00001	1.226 (1.165-1.291)	<0.00001
ALT, U/L	1.001 (0.999-1.004)	0.33698	1.001 (0.998-1.004)	0.62688
AST, U/L	1.009 (1.006-1.013)	<0.00001	1.003 (1.001-1.006)	0.02126
GGT, U/L	1.002 (1.000-1.003)	0.01270	1.001 (1.000-1.002)	0.02664
FPG, mmol/L	1.137 (1.066-1.212)	0.00009	1.103 (1.081-1.124)	<0.00001
2hPG, mmol/L	1.081 (1.059-1.104)	<0.00001	1.057 (1.045-1.069)	<0.00001
HbA1c, %	1.409 (1.297-1.532)	<0.00001	1.199 (1.162-1.237)	<0.00001
Fasting insulin, μU/mL	1.042 (1.033-1.050)	<0.00001	1.010 (1.006-1.015)	<0.00001
Smoking status				
Never smoker	Ref.	–	Ref.	–
Sometimes smoker	1.101 (0.837-1.447)	0.49168	1.231 (0.879-1.725)	0.22660
Regular smoker	1.308 (1.159-1.477)	0.00001	1.256 (1.067-1.480)	0.00623
Not recorded	1.082 (0.702-1.668)	0.72181	0.680 (0.328-1.409)	0.29988
Drinking status				
Never drinker	Ref.	–	Ref.	–
Sometimes drinker	1.037 (0.863-1.246)	0.69924	1.103 (0.860-1.416)	0.43995
Regular drinker	1.445 (1.226-1.705)	0.00001	1.524 (1.228-1.891)	0.00013
Not recorded	1.212 (0.774-1.898)	0.39970	0.909 (0.445-1.857)	0.79292
Exercise status				
No	Ref.	–	Ref.	–
Yes	0.588 (0.533-0.649)	<0.00001	0.680 (0.581-0.796)	<0.00001
Not recorded	0.921 (0.487-1.742)	0.79902	1.339 (0.605-2.966)	0.47191
Tumor				
Yes	Ref.	–	Ref.	–
No	1.066 (0.849-1.339)	0.58143	0.998 (0.743-1.341)	0.99034
Hypertension	1.793 (1.660-1.937)	<0.00001	1.693 (1.480-1.935)	<0.00001
Obesity	1.326 (1.200-1.466)	<0.00001	1.140 (1.003-1.295)	0.04434

Values are n (%) or mean ± SD or median (quartile).

BMI, body mass index; MAP, mean arterial pressure; WC, waist circumference; HC, hip circumference; SCr, serum creatinine; HDL-c, high-density lipoprotein cholesterol; LDL-c, low-density lipoprotein cholesterol; TC, total cholesterol; TG, triglycerides; ALT, alanine aminotransferase; AST, aspartate aminotransferase; GGT, gamma-glutamyl transferase; FPG, fasting plasma glucose; 2hPG, 2-hour plasma glucose; HbA1c, glycated hemoglobin; eGFR, estimated glomerular filtration rate; UACR, urinary albumin-to-creatinine ratio; CKD, chronic kidney disease. DM, Diabetes mellitus, Non-DM, No diabetes mellitus; HOMA-IR, homeostasis model assessment of insulin resistance.

### The results of multivariate analyses using the binary logistic regression model

We constructed three logistic regression models to explore the relationship between HOMA-IR and the risk of CKD in the DM and non-DM groups (**Table 4**). Among non-DM individuals, statistically significant positive associations between HOMA-IR and CKD risk were consistently demonstrated across all analytical models. Specifically, in the unadjusted model (Model I), each one-unit increment in HOMA-IR corresponded to a 4.9% elevation in the risk of CKD (OR = 1.049, 95%CI: 1.022–1.077). Upon incorporating solely demographic variables into the minimally adjusted model (Model II), a one-unit increment in HOMA-IR remained significantly associated with a 4.2% augmentation in CKD risk (OR = 1.042, 95%CI: 1.015–1.070). In the fully adjusted model (Model III), the risk of CKD increased by 3.7% (OR = 1.037, 95%CI: 1.010-1.066) for each unit increase in HOMA-IR. However, HOMA-IR was not significantly associated with the risk of CKD in the DM group (Model I: OR = 0.996, 95%CI: 0.958-1.035; Model II: OR = 0.984, 95%CI: 0.946-1.024; Model III: OR = 0.991, 95%CI: 0.952-1.032).

**Table 4:** Association between HOMA-IR and CKD in different models in patients with or without diabetes.

Variable	Model I (OR,95%CI, P)	Model II (OR,95%CI, P)	Model III (OR,95%CI, P)	Model IV (OR,95%CI, P)
All populations				
HOMA-IR	1.032 (1.010, 1.055) 0.00445	1.023 (1.000, 1.045) 0.04651	1.023 (1.000, 1.046) 0.04652	1.024 (1.001, 1.047) 0.04359
HOMA-IR Tertile				
T1	Ref.	Ref.	Ref.	Ref.
T2	1.076 (0.996, 1.161) 0.06166	1.179 (1.090, 1.275) 0.00004	1.180 (1.089, 1.277) 0.00005	1.207 (1.114, 1.309) <0.00001
T3	1.351 (1.255, 1.454) <0.00001	1.338 (1.242, 1.443) <0.00001	1.356 (1.254, 1.465) <0.00001	1.386 (1.281, 1.501) <0.00001
P for trend	<0.00001	<0.00001	<0.00001	<0.00001
Non-DM				
HOMA-IR	1.049 (1.022, 1.077) 0.00029	1.042 (1.015, 1.070) 0.00227	1.037 (1.010, 1.066) 0.00805	1.037 (1.009, 1.066) 0.00929
HOMA-IR Tertile				
T1	Ref.	Ref.	Ref.	Ref.
T2	1.111 (1.013, 1.218) 0.02541	1.232 (1.121, 1.354) 0.00002	1.220 (1.108, 1.343) 0.00005	1.241 (1.126, 1.368) 0.00001
T3	1.467 (1.342, 1.603) <0.00001	1.464 (1.338, 1.603) <0.00001	1.455 (1.325, 1.598) <0.00001	1.481 (1.346, 1.629) <0.00001
P for trend	<0.00001	<0.00001	<0.00001	<0.00001
DM				
HOMA-IR	0.996 (0.958, 1.035) 0.82828	0.984 (0.946, 1.024) 0.42312	0.991 (0.952, 1.032) 0.66711	0.996 (0.956, 1.037) 0.83291
HOMA-IR Tertile				
T1	Ref.	Ref.	Ref.	Ref.
T2	0.992 (0.864, 1.139) 0.90929	1.062 (0.922, 1.223) 0.40211	1.082 (0.937, 1.250) 0.28313	1.207 (1.114, 1.309) <0.00001
T3	1.126 (0.987, 1.285) 0.07688	1.103 (0.964, 1.261) 0.15232	1.147 (0.998, 1.319) 0.05386	1.386 (1.281, 1.501) <0.00001
P for trend	0.06019	0.15538	0.05456	0.01503

Model I, we did not adjust other covariants.

Model II, we adjusted age, sex, BMI.

Model III, was simultaneously adjusted for age, sex, BMI, ALT, AST, GGT, HDL-c, TG, smoking, drinking, exercise, hypertension, and tumor history.

Model IV, we simultaneously adjusted age (smooth), sex, BMI(Smooth), ALT(Smooth), AST(Smooth); GGT(Smooth), HDL-c(Smooth), TG(Smooth), smoking habits, drinking habits, working habits, hypertension, tumor.

OR, odds ratios; CI: confidence, Ref, reference; HOMA-IR, homeostasis model assessment of insulin resistance; Non-DM, No Diabetes mellitus; DM, Diabetes mellitus.

To determine whether insulin resistance was associated with proteinuria, decreased renal function, or both, we analyzed these relationships in diabetic and nondiabetic populations. In non-diabetic subjects, HOMA-IR was significantly associated with microalbuminuria (urinary albumin excretion rate > 30 mg/g) across all models (model III: OR 1.046, 95% CI 1.017-1.075, p = 0.00175)(**Supplementary Table 2**). However, no significant association was found in diabetic patients (Model III: OR 0.998, 95% CI 0.958-1.040, p = 0.94157). Regarding preserved eGFR < 60 ml/min/1.73 m², HOMA-IR showed no significant correlation in either diabetic patients (Model III: OR = 0.966, 95% CI 0.882-1.058, p = 0.46122) or non-diabetic individuals (Model III: OR = 1.005, 95%CI 0.939-1.076, p = 0.88015) (**Supplementary Table 3**).To determine if the association between insulin resistance and proteinuria was independent of reduced glomerular filtration, we analyzed individuals with normal renal function (eGFR≥60 ml/min/1.73 m²)(**Supplementary Table 4**). In this subgroup, the significant positive association between HOMA-IR and proteinuria in non-diabetic patients persisted (Model III: OR 1.044, 95% CI 1.014-1.074, p = 0.00346), while remaining non-significant in diabetic patients (Model III: OR 0.996, 95% CI 0.955-1.039, p = 0.85716). This strongly suggests that insulin resistance may be involved in the early stages of renal injury, prior to the decline in filtration function, which is the first stage of renal injury.

In addition, patients in the diabetes population were stratified according to comprehensive glycemic control status (**Supplementary Table 5**).The patients were divided into well-controlled group (FPG<7.0 mmol/L, 2hPG<11.1 mmol/L, HbA1c<6.5%) and poorly-controlled group (FPG≥7.0 mmol/L, 2hPG≥11.1 mmol/L, HbA1c≥6.5%). The results suggest that we did not observe a significant association between HOMA-IR and the risk of CKD in people with diabetes, regardless of glycemic control.

### Sensitivity analysis

To confirm the robustness of the results of this study, we performed a series of sensitivity analyses by transforming HOMA-IR into tertiles based categorical variables and incorporating them into the model. **Table 4** shows the association between HOMA-IR tertiles and CKD risk in people with or without diabetes. In the non-DM group, compared with the lowest tertile group, the OR for CKD in the second and third tertile groups was 1.111 (95% CI, 1.013-1.218) and 1.467 (95% CI, 1.342-1.603), respectively, in the unadjusted model (Model I). The associations remained significant after adjustment for age, sex, and BMI (Model II). In the fully adjusted model (Model III), the OR for the second and third quantile groups were 1.220 (95% CI, 1.108-1.343) and 1.455 (95% CI, 1.325-1.598), respectively (P for trend <0.0001). In the DM population, HOMA-IR was not associated with CKD risk in either unadjusted (Model I), minimally adjusted (Model II), or fully adjusted (Model III) models. These results suggest a potential nonlinear relationship between HOMA-IR and CKD, as the trend of effect sizes was not exactly the same when HOMA-IR was classified. Further analyses using GAM included continuous covariates as curves. The results of Model IV were consistent with those of the fully adjusted model (Model III) regardless of whether the participants had diabetes (Non-DM group: OR = 1.037; 95%CI, 1.009-1.066; P<0.05; DM group: OR = 0.996; 95% CI, 0.956-1.037; P>0.05).

### The nonlinearity addressed by the generalized additive model

**Figure 6:**
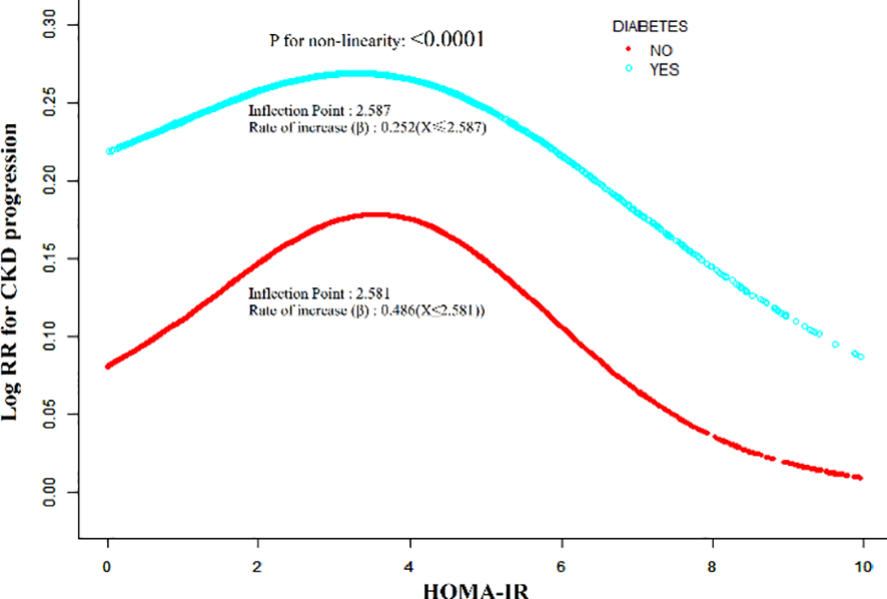
Non-linear relationship and two-piecewise regression equations between HOMA-IR and CKD risk. Smooth curve fitting generated by generalized additive models (GAM) showing the N-shaped relationship between HOMA-IR and log relative risk (Log RR) of CKD. Solid lines represent the estimated risk, and shaded areas represent 95% confidence intervals. The models were adjusted for age, sex, BMI, ALT, AST, GGT, HDL-c, TG, smoking, drinking, exercise, hypertension, and tumor history. Equations: The behavior is mathematically defined by two-piecewise logistic regression. For Non-DM (Inflection point = 2.581):β=0.486 (when HOMA-IR≤ 2.581) and β=−0.250 (when HOMA-IR > 2.581). For DM (Inflection point = 2.587): β=0.252 (when HOMA-IR≤ 2.587) andβ=−0.164 (when HOMA-IR > 2.587). The steeper slope (β=0.486) in the Non-DM group indicates a more rapid rate of CKD risk increase per unit of HOMA-IR before the threshold.

Generalized additive models and smooth curve fitting showed a nonlinear relationship (N-type relationship) between HOMA-IR and CKD risk in both the non-diabetic and diabetic groups (**Figure 6**). Drawing upon the results of a sensitivity analysis, the dataset was subjected to fitting procedures utilizing a standard logistic regression model, with the optimal model fit subsequently determined and selected on the basis of statistical significance (P < 0.05) as evaluated through the application of a log-likelihood ratio test. Subsequently, we 379 implemented a two-piecewise binary logistic regression model and found the turning point at 2.581–380 in the non-diabetic group and 2.587 in the diabetic group. Based on the two-piecewise logistic regression equations (Logit(P)=β0+β1×X), we further quantified and compared the rate of increase (slope β, derived from ln(OR)) between the two populations. In the non-DM group, the OR for below the threshold was 1.625 (95% CI, 1.518-1.740), yielding a steep positive slope (β=0.486). The OR for above the threshold was 0.779 (95% CI, 0.738-0.822;β=−0.250). In the DM group, the OR for being below the threshold was 1.286 (95% CI, 1.167-1.417), corresponding to a gentler slope (β=0.252), and the OR for being above the threshold was 0.849 (95% CI, 0.792-0.910; β=−0.164). Notably, by comparing these equations, the mathematical rate of increase in CKD risk driven by HOMA-IR before the inflection point is nearly twice as fast in non-diabetic individuals (β=0.486) compared to diabetic patients (β=0.252) (**Table 5**).

**Table 5:** The results of two-segment Logistic regression models for patients with or without diabetes.

Logistic regression	Model I (OR, 95%CI, P)	
	Non-DM	DM
Fitting model by standard logistic regression	1.037 (1.010, 1.066) 0.0081	0.991 (0.952, 1.032) 0.6671
Fitting model by two-piecewise logistic regression		
Inflection point of the HOMA-IR	2.581	2.587
≤Inflection point	1.625 (1. 518, 1.740) <0.0001	1.286 (1.167, 1.417) <0.0001
>Inflection point	0.779 (0.738, 0.822) <0.0001	0.849 (0.792, 0.910) <0.0001
P for log-likelihood ratio test	<0.001	<0.001

We adjusted simultaneously age, sex, BMI, ALT, AST, GGT, HDL-c, TG, smoking habits, drinking habits, exercise status, hypertension, tumor.

OR, odds ratios; CI, confidence; Ref, reference; HOMA-IR, homeostasis model assessment of insulin resistance; Non-DM, No Diabetes mellitus; DM, Diabetes mellitus.

In addition, among patients with diabetes, analyses stratified according to glycemic control showed consistent nonlinear patterns (**Supplementary Figure 2**, **Supplementary Table 6**). The crossing point was 2.588 in the well-controlled group and 1.003 in the poorly-controlled group. In the well-controlled group, the OR for below the threshold was 1.373 (95% CI, 1.202 to 1.569), and the OR for above the threshold was 0.720 (95% CI, 0.643-0.807). In the group with poor glycemic control, the odds ratio for being below the threshold was 3.917 (95% CI,1.628 to 9.425) and the odds ratio for being above the threshold was 1.002 (95% CI,0.941 to 1.067).

### The results of subgroup analyses

Subgroup analyses were subsequently performed to systematically evaluate the potential confounding effects of relevant covariates on the association between HOMA-IR and CKD among individuals stratified by diabetes status. A comprehensive set of stratification variables — encompassing age, sex, BMI, hypertension, obesity, cancer, occupational status, smoking habits, and alcohol consumption status — were incorporated into the analytical framework to thoroughly investigate the directional trends and magnitude of effect sizes across these respective subgroups. **Supplementary Table 7** shows that none of the above potentially confounding variables affected the relationship between HOMA-IR and CKD risk regardless of whether the participants had diabetes, and we did not observe any interaction, indicating that our results were very robust.

### Interaction of diabetes status (non-DM and DM) on the relationship between HOMA-IR and risk of CKD

To investigate the interaction effect of diabetes status (non-DM and DM) on the relationship between HOMA-IR and CKD risk, a stratified regression analysis was conducted, and interaction models were constructed. The analysis included unadjusted models (Crude) and progressively adjusted models (Model I and Model II), which evaluated the impact of HOMA-IR on CKD risk and tested the interaction effect of diabetes status. In the non-DM group, HOMA-IR was consistently and significantly associated with an increased risk of CKD across all models (Crude model: OR = 1.049, 95% CI: 1.022–1.077, P = 0.0003; Model I: OR = 1.042, 95% CI: 1.015–1.070, P = 0.0021; Model II: OR = 1.042, 95% CI: 1.015–1.071, P = 0.0024). This indicates that in the non-DM population, each unit increase in HOMA-IR was associated with a 4.2%–4.9% increase in CKD risk. However, in the DM group, no significant association was observed between HOMA-IR and CKD risk (Crude model: OR = 0.996, 95% CI: 0.958–1.035, P = 0.8283; Model I: OR = 0.982, 95% CI: 0.943–1.021, P = 0.3562; Model II: OR = 0.982, 95% CI: 0.944–1.023, P = 0.3859). The interaction test results showed that diabetes status significantly modified the relationship between HOMA-IR and CKD risk (All P for Interaction <0.05; **Table 6**).

**Table 6:** Interaction analysis between HOMA-IR and CKD risk stratified by diabetes status.

Model	Non-DM (OR, 95% CI, P-value)	DM (OR, 95% CI, P-value)	P for Interaction
Model I	1.049 (1.022–1.077) 0.0003	0.996 (0.958–1.035) 0.8283	0.0272
Model II	1.042 (1.015–1.070) 0.0021	0.982 (0.943–1.021) 0.3562	0.0132
Model III	1.042 (1.015–1.071) 0.0024	0.982 (0.944–1.023) 0.3859	0.0149

Model I. we did not adjust other covariants.

Model II. we adjusted age, sex, BMI.

Model III was simultaneously adjusted for age, sex, BMI, ALT, AST, GGT, HDL-c, TG, smoking, drinking, exercise, hypertension, and tumor history.

OR, odds ratios; CI, confidence; Ref, reference; HOMA-IR, homeostasis model assessment of insulin resistance; Non-DM, No Diabetes mellitus; DM, Diabetes mellitus.

## Discussion

To the best of our knowledge, this is one of the largest cross-sectional studies to comprehensively investigate the differential association between HOMA-IR and CKD risk in both diabetic and non-diabetic Chinese populations. The primary contribution and uniqueness of this study lie in two aspects. First, we robustly demonstrated that elevated HOMA-IR acts as an independent predictor of CKD predominantly in non-diabetic individuals, underscoring its clinical diagnostic value as an early warning biomarker prior to the onset of clinical diabetes. Second, our study uniquely characterized a non-linear, N-shaped relationship between HOMA-IR and CKD risk across different metabolic states, providing specific threshold values (inflection points at 2.581 for non-diabetics and 2.587 for diabetics). This offers a more nuanced understanding of insulin resistance in renal pathophysiology than previous linear models. Ultimately, these findings highlight the necessity of tailoring risk assessment and individualized intervention strategies for CKD based on the patient’s baseline diabetes status.

The prevalence of CKD in our study population was 16.09%, which is comparable to the previous estimate of 10.8% to 16.9% in the Chinese population (48). However, this rate is slightly higher than the 13.4% global prevalence reported in the meta-analysis (49). In addition, the calculated prevalence of CKD in our study was 25.07% and 13.51% in diabetic and non-diabetic patients, respectively. We review the literature and find that this rate is consistent with the results of a previous study, which showed (50) a prevalence of CKD of 16.57% and 9.57% in diabetic and non-diabetic patients, respectively. All the above results indicate that the prevalence of CKD is higher in diabetic patients than in non-diabetic patients. Furthermore, the observed prevalence of CKD among diabetic patients in the present study was markedly elevated relative to that recorded in their non-diabetic counterparts (31.94% vs. 16.57%), a disparity that may be attributable to several underlying key contributing factors.

First, the demographic characteristics of the study population, including a relatively high BMI (24.64 ± 3.69 kg per square meter), a high proportion of persons with hypertension (60.31%), and a high proportion of metabolic risk factors, may have influenced the observed prevalence. In addition, methodological discrepancies in the defining criteria applied to CKD — most notably the utilization of albuminuria as a primary diagnostic marker in the present study — may have substantially contributed to the relatively elevated prevalence observed, given that albuminuria serves as a highly sensitive and clinically recognized indicator of early-stage renal damage. These population-specific demographic and clinical characteristics, in conjunction with considerable heterogeneity in study design methodologies across the included studies, may collectively account for and elucidate the discrepancies detected in the reported prevalence estimates of CKD.

Currently, there has been increasing attention to the complex relationship between HOMA-IR and CKD (7, 24, 51, 52). This relationship is characterized as bidirectional, with CKD presenting a low-grade inflammatory state that can induce insulin resistance, which in turn accelerates the deterioration of renal function (6, 7). Synthesizing the current literature reveals a multi-pathway mechanism by which early hyperinsulinemia drives renal injury, providing a biological rationale for our findings. Rather than a single isolated trigger, insulin resistance initiates a cascade of interlocking pathological events. On one hand, compensatory hyperinsulinemia stimulates the production of reactive oxygen species (ROS), precipitating profound endothelial dysfunction and disrupting normal vascular permeability (43). On the other hand, it aberrantly activates the systemic and intrarenal renin-angiotensin-aldosterone system (RAAS), which mechanically forces the kidneys into a state of glomerular hyperfiltration (53). Based on these intertwined pathways, it can be logically deduced that the initial stages of insulin resistance uniquely precipitate structural renal damage—specifically podocyte effacement and subsequent proteinuria—long before a frank decline in glomerular filtration rate (eGFR) or overt hyperglycemia manifests (54, 55). This inductive deduction perfectly aligns with our clinical observation that elevated HOMA-IR independently and predominantly predicts early CKD risk (notably albuminuria) in the non-diabetic population.

The results of this study show a significant positive correlation between HOMA-IR and CKD risk, which is generally consistent with previous studies. A prior investigation leveraging NHANES data (56) demonstrated that individuals belonging to the highest HOMA-IR quartile exhibited a substantially elevated CKD risk, specifically 2.65 times greater (95% CI: 1.25–5.62) in comparison to those with normal HOMA-IR values. Corroborating these findings, a cross-sectional investigation conducted by Zammit et al. (42) further established that HOMA-IR was both statistically significant and independently predictive of CKD occurrence (OR = 1.02, 95% CI: 1.00–1.05). In a population of 5,232 individuals aged 40 and older without baseline CKD, HOMA-IR showed the strongest association with the risk of CKD incidence compared to other metabolic indicators in all Cox regression models (HR = 1.61, 95%CI: 1.10-2.34) (31). This indicates that the association between HOMA-IR and CKD is robust and independent.

However, some studies have also reported that HOMA-IR was not significantly associated with CKD risk (24,29,58). In a multicenter prospective cohort study of 1,883 non-diabetic participants with chronic renal insufficiency (24), HOMA-IR was not significantly associated with CKD progression (HR = 1.01,95%CI: 0.90-1.14). Corroborating evidence from a Chinese investigation (28) demonstrated that multivariate logistic regression analysis revealed a statistically significant association between HOMA-IR values and the prevalence of CKD exclusively among male participants, whereas no such significant association was observed in their female counterparts (Male: OR = 1.21; 95%CI 1.14–1.28, p ≤ 0.001; Female: OR = 1.01; 95%CI 0.99–1.02, p = 0.38). Consistent with these findings, a separately conducted Turkish study (57) further reported that elevated HOMA-IR levels were independently associated with a significant decline in eGFR among male subjects, a relationship that did not reach statistical significance in female subjects.

The apparent differences in the literature can be attributed to several factors. First, differences in study design and analytical methods may lead to inconsistent results. For example, cohort and cross-sectional studies have inherent limitations in causal inference and time-series analysis, which may result in different interpretations of the association between HOMA-IR and CKD risk. Second, differences in the characteristics of the included populations may also influence the findings. Some studies focus on high-risk populations (such as those with obesity or metabolic syndrome), while others are based on the general population, which may lead to less comparable results. The participants in this study generally had higher rates of age, BMI, and hypertension, factors that often coexist with metabolic syndrome and have been shown to be common risk factors for CKD (58–61). Therefore, if these factors are not adequately adjusted, the independent role of HOMA-IR may be masked. Finally, considering the complexity of pathological mechanisms, HOMA-IR may affect CKD risk through different mechanisms in diabetic and non-diabetic populations. In diabetic patients, HOMA-IR may serve as an “intermediate mediator” of hyperglycemia-related renal injury, while in non-diabetic populations, elevated HOMA-IR may reflect the effects of obesity, chronic inflammation, or uremic toxin accumulation (22). which may accelerate the occurrence and progression of CKD by increasing oxidative stress, endothelial dysfunction, or renal fat deposition.

In addition, we grouped participants based on diabetes status and found a significant difference in the correlation between HOMA-IR and CKD in diabetic and non-diabetic populations. In the non-diabetic group, after adjusting for confounding factors, the risk of CKD significantly increased with higher HOMA-IR levels (OR = 1.037, 95% CI: 1.010–1.066, P<0.05). However, in the diabetic group, there was no significant correlation between the two (OR = 0.991, 95% CI: 0.952–1.032, P = 0.6671). Our results are consistent with two previous studies. A community-based prospective cohort study in Korea (23) indicated that in the non-diabetic population, high baseline HOMA-IR is an independent risk factor for CKD (OR = 1.49, 95% CI: 1.12–1.98, P = 0.01). A national cross-sectional survey (27) in China reported a significant positive correlation between HOMA-IR and the prevalence of CKD in non-diabetic group (OR = 1.549, 95%CI: 1.079-2.223, P<0.05). However, previous studies have also reported opposing results.

By grouping participants based on diabetes status, we identified a stark contrast in the diagnostic utility of HOMA-IR: while HOMA-IR exhibited a significant positive correlation with CKD risk in the non-diabetic cohort, it lost its independent predictive value in the diabetic group. This differential association can be logically inferred by integrating findings from recent cross-sectional, case-control, and longitudinal studies. Synthesizing current evidence reveals that while early insulin resistance triggers renal injury, the diagnostic value of HOMA-IR dynamically changes as diabetes progresses. From a pathophysiological perspective, established type 2 diabetes is characterized by progressive pancreatic β-cell exhaustion, leading to an absolute or relative decline in fasting insulin levels (30). Consequently, the mathematically derived HOMA-IR value may misleadingly appear to ‘improve’ or plateau, failing to accurately reflect the true severity of tissue-level insulin resistance. Furthermore, several longitudinal cohort studies have demonstrated that in diabetic populations, structural renal damage is overwhelmingly driven by prolonged hyperglycemia (29), the accumulation of advanced glycation end products (AGEs) (62), and the concurrent use of antidiabetic medications (e.g., exogenous insulin, metformin, or SGLT2 inhibitors) (63, 64). These downstream pathological factors complexify the disease trajectory and dilute the independent diagnostic value of HOMA-IR. Collectively, we can deduce that HOMA-IR serves as a highly sensitive early screening marker for CKD in non-diabetic individuals, yet its standalone diagnostic utility is significantly confounded and limited once overt diabetes is established.

Furthermore, our study found a nonlinear relationship between HOMA-IR and CKD risk in patients with and without diabetes. Such a finding reflects a degree of complexity that surpasses the simple linear associations reported in certain earlier investigations (23, 31, 61), and the delineation of this nonlinear pattern may offer a plausible explanation for a number of apparently conflicting findings previously documented in the existing literature. A piecewise logistic regression model was applied in the present study to delineate the underlying N-shaped dose-response relationship between HOMA-IR and CKD risk. Following comprehensive adjustment for potential confounding variables, the threshold inflection points were separately identified at 2.587 and 2.581 for the diabetic and non-diabetic subpopulations, respectively. The results showed that, regardless of diabetes status, when HOMA-IR is below the threshold, the risk of developing CKD significantly increases with higher HOMA-IR levels (≤2.587: OR = 1.286, 95% CI: 1.167–1.417; ≤2.581: OR = 1.625, 95% CI: 1.518–1.740; P < 0.0001). Conversely, when HOMA-IR was above the threshold, the risk of CKD was significantly reduced with the increase of HOMA-IR level (>2.587: OR = 0.849, 95% CI: 0.792–0.910; >2.581: OR = 0.779, 95% CI: 0.738–0.822; P < 0.0001). These results indicate that the risk of CKD is highest when HOMA-IR values are near the inflection points, regardless of diabetes status.

Crucially, as reflected by our piecewise regression equations, the mathematical rate of increase in CKD risk before the inflection point differs remarkably between the two populations. The slope of risk escalation in non-diabetics (β=0.486) is substantially steeper than in diabetics (β=0.252). This steeper rate of increase strongly suggests that the healthy kidney is highly sensitive and vulnerable to the initial onset of hyperinsulinemia. In non-diabetics, early insulin resistance acts as an aggressive primary hit to the glomerulus. In contrast, in established diabetics, the kidneys are simultaneously subjected to multiple confounding insults—such as glucotoxicity, advanced glycation end-products, and compensatory renal hypertrophy—which may blunt or dilute the isolated rate of injury driven solely by fluctuating HOMA-IR.

However, this finding is less consistent with the results of a previous study (65), which recruited 10,660 participants, 15.42% with CKD, and found a U-shaped relationship between HOMA-IR and CKD prevalence with an inflection point of 2.5. When HOMA-IR was <2.5, the risk of CKD decreased rapidly with decreasing HOMA-IR levels; when HOMA-IR was ≥2.5, the risk of CKD increased rapidly with increasing HOMA-IR levels (OR = 1.02, 95% CI: 1.01-1.03). Although the mechanisms underlying the relationship between HOMA-IR levels and CKD development are not yet clear, several explanations can account for the differing relationships between HOMA-IR and CKD prevalence before and after the inflection point. (1) When HOMA-IR values exceed the inflection point, their relative contribution to CKD risk may weaken due to the presence of multiple CKD risk factors simultaneously. Conversely, when HOMA-IR values remain below the identified inflection point, comparatively diminished levels of additional risk factors — notably BMI, circulating blood lipids, and the proportion of concurrent metabolic disorders — may attenuate their respective confounding influences, consequently magnifying the proportional contribution of HOMA-IR to the overall risk of CKD development. (2) In contradistinction to the present study, the aforementioned investigations failed to account for the potential modulatory effects of ALT, AST, GGT, occupational status, and familial history of cancer on the underlying association between HOMA-IR and CKD within their covariate adjustment frameworks. Nevertheless, accumulating evidence from previously published literature has consistently identified and confirmed these aforementioned variables as being meaningfully implicated in either CKD pathogenesis or HOMA-IR dysregulation (66, 67). (3) The human body may develop compensatory protective mechanisms under long-term high insulin resistance, mitigating kidney damage by upregulating certain protective factors (68, 69). Additionally, the most severe cases among patients with high HOMA-IR may have already progressed to end-stage renal disease or death and dropped out of the study cohort, resulting in the observed high HOMA-IR patients being relatively “healthier” individuals. Alternatively, patients with high HOMA-IR may have received aggressive therapeutic interventions, which effectively reduced their CKD risk. (4) The differences may also be related to variations in renal function. Multiple studies have shown that the association between HOMA-IR and CKD risk varies across different stages of CKD (10, 24).

This unexpected resilience in the diabetic cohort can be profoundly explained through the lens of early clinical intervention and the reversibility of albuminuria. Clinically, a UACR of 30–300 mg/g represents microalbuminuria, a critical window during which early CKD is still reversible. Because patients with established diabetes are subjected to stringent, guideline-directed medical therapy and routine microalbuminuria screening (47), those entering the 30–300 mg/g stage are highly likely to receive aggressive nephroprotective interventions (such as ACE inhibitors or Angiotensin II Receptor Blockers). These treatments effectively halt or even reverse the progression of CKD, masking the natural pathophysiological damage driven by high HOMA-IR. Consequently, diabetic participants mathematically present a “flatter” risk trajectory and appear paradoxically healthier. Conversely, non-diabetic individuals with hyperinsulinemia lack routine UACR screening; thus, their renal injury progresses unabatedly through the 30–300 mg/g reversible window into irreversible structural damage (>300 mg/g), explaining the much steeper rate of CKD risk escalation in the non-diabetic population.

The subsequent attenuation or slight decrease in risk observed at higher HOMA-IR levels likely reflects a complex interplay of multiple factors, rather than a diminished pathological impact of severe insulin resistance per se. First, survival bias may be operative, as individuals with severe insulin resistance who develop significant renal dysfunction may have been systematically excluded from our cross-sectional sample due to prior progression to end-stage renal disease or premature mortality. Second, individuals with recognized severe insulin resistance are more likely to have received pharmacological interventions — such as metformin or SGLT2 inhibitors — that may attenuate kidney damage despite the persistence of underlying metabolic abnormalities (63, 64). Third, physiological compensatory mechanisms may be activated at extreme degrees of insulin resistance, including the upregulation of alternative metabolic pathways or adaptive renal autoregulatory responses that transiently stabilize kidney function in the setting of ongoing metabolic stress (53).

This complex non-linear relationship helps explain inconsistencies in previous literature, where studies examining different ranges of insulin resistance or different populations may capture different segments of this N-shaped curve, leading to apparently contradictory conclusions about the association between insulin resistance and CKD (65). Our comprehensive modeling approach reveals that both positive associations and null findings in previous research may be reconciled within this more nuanced non-linear framework, representing different portions of the same underlying biological relationship that transcends diabetes status.

Our findings strongly suggest that insulin resistance is the primary driver of proteinuria formation — an early indicator of kidney damage — and that proteinuria typically precedes any measurable decline in glomerular filtration rate. This pattern is consistent with the well-established natural history of most progressive kidney diseases, in which proteinuria emerges before a detectable reduction in GFR (70). Our results further indicate that insulin resistance may be particularly important in the earliest stages of renal functional impairment, manifesting as glomerular barrier dysfunction and protein leakage, especially in non-diabetic patients. This is supported by plausible biological mechanisms, whereby insulin resistance promotes glomerular hyperfiltration, endothelial dysfunction, oxidative stress, and podocyte injury — factors that collectively disrupt the glomerular filtration barrier and present clinically as proteinuria (55). In contrast, the pathways responsible for GFR decline — including chronic renin-angiotensin-aldosterone system (RAAS) activation and tubulointerstitial fibrosis — typically evolve gradually over time, which may account for the weak or inconsistent cross-sectional associations observed between HOMA-IR and reduced GFR.

This finding identifies insulin resistance as a marker of early, potentially reversible kidney damage, suggesting that interventions targeting insulin sensitivity could prevent progression to overt CKD if implemented before significant eGFR decline occurs (71). This finding has several important clinical implications. Non-diabetic individuals with elevated insulin resistance represent a critical window for intervention before diabetes onset, and unlike diabetic patients who may already have established metabolic damage, non-diabetic individuals with insulin resistance represent a population where lifestyle interventions could be particularly effective. Additionally, HOMA-IR could serve as an early screening tool in pre-diabetic populations to identify those at highest risk for kidney damage, offering a cost-effective approach to early detection.

Our findings suggest that targeted interventions to improve insulin sensitivity in non-diabetic individuals with elevated HOMA-IR could represent a novel primary prevention strategy for CKD. Such an approach would encompass three main components: lifestyle modifications — including dietary optimization, regular exercise, and weight management — to improve insulin sensitivity (72), early pharmacological intervention with agents such as metformin or SGLT2 inhibitors in high-risk non-diabetic individuals (73, 74), and routine albuminuria monitoring in non-diabetic patients with elevated HOMA-IR (71). Collectively, these interventions could potentially prevent the progression from early glomerular damage to overt CKD, representing a paradigm shift from managing established disease toward preventing its onset.

Our findings have important clinical implications, particularly with respect to the significant differential relationship between HOMA-IR and CKD risk across diabetes states. This finding provides a new perspective to further understand the mechanism of HOMA-IR in the development and progression of CKD and highlights the importance of stratified management according to diabetes status. This is particularly valuable given the continuing worldwide increase in the prevalence of CKD and its associated morbidity and mortality. The establishment of HOMA-IR as a viable therapeutic intervention target holds considerable promise for the development of more refined and individualized prevention and treatment strategies in the foreseeable future, ultimately contributing to a reduction in the overall disease burden attributable to CKD and to the enhancement of long-term clinical prognosis among affected patient populations.

This study has several notable strengths that enhance the reliability and generalizability of our findings. First, our analyses were based on a large, multicenter sample (n = 32,055), which provided robust representation of the Chinese population, ensured adequate statistical power, and strengthened the external validity of our results. Second, we employed a comprehensive and methodologically rigorous statistical framework. Specifically, the application of GAM enabled us to detect and characterize the nonlinear relationship between HOMA-IR and CKD risk — a feature that conventional linear regression models would have failed to capture. Third, methodological rigor was further demonstrated through careful consideration of potential confounders: we adjusted extensively for multiple demographic, clinical, and lifestyle factors, and conducted stratified analyses to examine potential effect modification. Together, this comprehensive analytical approach, combined with the multicenter study design, substantially strengthened the internal validity of our findings and provided robust evidence for the observed associations.

Several inherent limitations of the present study warrant careful consideration and acknowledgment. Foremost, it must be recognized that the current investigation was conducted as a cross-sectional analysis derived from a secondary data source based upon a publicly accessible database; as such, the cross-sectional nature of the study design fundamentally precludes the establishment of any definitive temporal sequence or causal inference regarding the relationship between insulin resistance and the progression trajectories of kidney disease. Therefore, in order to intervene participants before kidney function decline to develop CKD, our future research will try to dig deeper into this database and related databases, and propose a longitudinal study framework, such as a time series study with longitudinal tracking of albuminuria and eGFR. Second, our original definition of chronic kidney disease as a combination of proteinuria (urinary albumin excretion rate ≥30 mg per gram) and a reduced estimated glomerular filtration rate (< 60 ml per minute per 1.73 m2 of body-surface area) may have obscured associations between specific phenotypes. In the future, we will focus on using longitudinal multicenter study to establish the time-series relationship and verify the predictive value of HOMA-IR for CKD development. Biochemical parameters and HOMA-IR values were measured repeatedly to better reflect the characteristics of long-term changes. Mmultiple measurements would allow more accurate assessment of chronic exposure and better delineation of metabolic trajectories. Third, despite the incorporation of extensive adjustment for a broad range of potential confounding variables, the possibility of residual confounding cannot be entirely excluded from consideration. Fourth, because CKD was not analyzed by stage, we could not gain insight into the specific role of HOMA-IR in different stages of CKD progression. There may be specific patterns of insulin resistance and risk associations across CKD stages, and the lack of such stratified analyses may mask important stage-specific features. Fifth, The present study was confined to a Chinese demographic cohort, a characteristic that may inherently constrain the broader applicability and generalizability of the observed findings to other ethnically distinct populations. Given that there may be significant differences in metabolic profiles and CKD risk factors between ethnic groups, future validation of these findings in other ethnic populations and establishment of ethnic-specific threshold criteria are needed. Sixth, we must acknowledge that in diabetic patients who use insulin to lower blood glucose levels, the use of exogenous insulin may interfere with the determination of endogenous plasma insulin, thereby affecting the validity of the insulin resistance index and possibly biasating the corresponding statistical results.

Future multicenter prospective cohort studies will employ innovative multidimensional integrative approaches to address current limitations. Treatment selection bias will be reduced by adjusting for confounding factors, conducting subgroup analyses, and incorporating insulin treatment plans (dose, type, and timing) as key covariates. We will develop a dual assessment system combining C-peptide (75) and standard insulin measurements that leverages C-peptide’s specificity for endogenous insulin secretion while avoiding exogenous insulin interference, leading to a validated modified HOMA-Cp equation (76).

## Conclusion

In conclusion, this study showed a significant difference between HOMA-IR and CKD risk in people with and without diabetes in Chinese population. In the Non-DM group, the risk of CKD increased significantly with the increase of HOMA-IR. However, there was no significant correlation between them in DM group. In addition, the nonlinear association between HOMA-IR and risk for CKD in patients with and without diabetes was noted. The inflection point was 2.581 in the Non-DM group and 2.587 in the DM group. Below this threshold, CKD risk increased significantly with increasing HOMA-IR regardless of diabetes status. Meanwhile, above the threshold, the risk of CKD decreased significantly with the increase of HOMA-IR level. This study provides a reference for future CKD prevention in populations with different diabetes status. Adjusting HOMA-IR may be a new therapeutic direction for reducing the risk of CKD.

## Data Availability

The datasets presented in this study can be found in online repositories. The names of the repository/repositories and accession number(s) can be found below: Data could be downloaded from the PLOS ONE database (https://journals.plos.org/plosone/).
