# Supplementary material for: Relationship of HOMA-IR with chronic kidney disease in diabetic and non-diabetic Chinese populations: findings from the REACTION study
Source: Front Endocrinol (Lausanne). 2026 Apr 21;17:1707947. doi: 10.3389/fendo.2026.1707947 (PMC13138936; doi:10.3389/fendo.2026.1707947)
Supplement: Supplementary file 1 [file DataSheet1.docx]

**Insulin resistance (HOMA-IR) and risk of chronic kidney disease (CKD) in people with and without diabetes: a large cross-sectional study in a Chinese population**

**Running title：HOMA-IR and CKD risk in people with and without diabetes**

**Yuheng Liao^#1,2^ , Mijie Guan^#1,3,4^, Qijun Wan^*1,3^ , Haiying Song^*1,2,3^, Haofei Hu^*1,3^**

^1^Department of Nephrology, Shenzhen Second People’s Hospital, Shenzhen 518000, Guangdong Province, China

^2^School of Medicine Shenzhen University, Shenzhen 518000, China

^3^Department of Nephrology, The First Affiliated Hospital of Shenzhen University, Shenzhen 518000, Guangdong Province, China

^4^Department of Vascular Access Center, The First Affiliated Hospital of Jinan University, Jinan University, Guangzhou 510630, China.

***Corresponding author**

**Qijun Wan**

Department of Nephrology,

Shenzhen Second People’s Hospital,

No.3002 Sungang Road, Futian District,

Shenzhen 518000,

Guangdong Province,

China

Tel:+86-755-83366388

E-mail: yiyuan2224@sina.com

***Corresponding author**

**Haiying Song**

Department of Nephrology,

Shenzhen Second People’s Hospital,

No.3002 Sungang Road, Futian District,

Shenzhen 518000,

Guangdong Province,

China

Tel:+86-755-83366388

E-mail: [haiyingsong2024@126.com](mailto:haiyingsong2024@162.com)

***Corresponding author**

**Haofei Hu**

Department of Nephrology,

Shenzhen Second People’s Hospital,

No.3002 Sungang Road, Futian District,

Shenzhen 518000,

Guangdong Province,

China

Tel:+86-755-83366388

E-mail: [huhaofei0319@126.com](mailto:huhaofei0319@126.com)

**Table S1. Collinearity diagnostics steps.**

| Variable | VIF  Step 1 | Step 2 |
| --- | --- | --- |
| Gender | 1.7 | 1.7 |
| Age(years) | 1.4 | 1.4 |
| BMI (kg/m2) | 1.2 | 1.2 |
| ALT(U/L) | 3.3 | 3.2 |
| AST(U/L) | 3.2 | 3.2 |
| GGT (U/L) | 1.3 | 1.3 |
| HDL-c(mmol/L) | 3.2 | 1.4 |
| LDL-c(mmol/L) | 11.7 | 1.2 |
| TG(mmol/L) | 2.7 | 1.2 |
| TC(mmol/L) | 17.2 | NA |
| Hypertension | 1.2 | 1.2 |
| Tumor | 1.0 | 1.0 |
| Smoking habits | 1.4 | 1.4 |
| Drinking habits | 1.3 | 1.3 |
| Exercise status | 1.3 | 1.3 |

Abbreviations: BMI, body mass index; HDL-c, high-density lipoprotein cholesterol; LDL-c, low-density lipoprotein cholesterol; TC, total cholesterol; TG, triglycerides; ALT, alanine aminotransferase; AST, aspartate aminotransferase; GGT, gamma-glutamyl transferase;

Note 1:VIF: variance inflation factor; VIF = 1/(1-R^2^).

Note 2: The variables with VIF>5 will be regarded as collinear variables and cannot be included in the multiple regression model.

**Table S2. The relationship between HOMA-IR and UACR > 30mg/g in diabetes and non-diabetic patients under different models**

| **Variable** | **Model I (OR,95%CI, P)** | **Model II(OR,95%CI, P)** | **Model III (OR,95%CI, P)** |
| --- | --- | --- | --- |
| **DM** | | | |
| **HOMA-IR** | 1.000 (0.961, 1.040) 0.98400 | 0.990 (0.951, 1.031) 0.63524 | 0.998 (0.958, 1.040) 0.94157 |
| **Non-DM** | | | |
| **HOMA-IR** | 1.056 (1.028, 1.085) 0.00008 | 1.049 (1.021, 1.078) 0.00057 | 1.046 (1.017, 1.075) 0.00175 |

Model I: we did not adjust other covariants;

Model II: we adjusted age, sex, BMI;

Model III: we adjusted age, sex, BMI, ALT, AST, GGT, HDL-c, TG, smoking habits, drinking habits , working habits, hypertension, tumor;

Model IV: we adjusted age (smooth), sex, BMI(Smooth), ALT(Smooth), AST(Smooth); GGT(Smooth), HDL-c(Smooth), TG(Smooth), smoking habits, drinking habits, working habits, hypertension, tumor;

OR, odds ratios; CI: confidence, Ref: reference; HOMA-IR, homeostasis model assessment of insulin resistance; Non-DM: No Diabetes mellitus; DM: Diabetes mellitus;

**Table S3. Relationship between HOMA-IR and eGFR<60 mL/min/1.73m²in diabetes and non-diabetic patients under different models**

| **Variable** | **Model I (OR,95%CI, P)** | **Model II(OR,95%CI, P)** | **Model III (OR,95%CI, P)** |
| --- | --- | --- | --- |
|  |  | **DM** |  |
| **HOMA-IR** | 0.976 (0.894, 1.066) 0.59168 | 0.983 (0.901, 1.072) 0.69187 | 0.966 (0.882, 1.058) 0.46122 |
|  |  | **Non-DM** |  |
| **HOMA-IR** | 1.013 (0.947, 1.083) 0.71065 | 1.020 (0.955, 1.090) 0.55058 | 1.005 (0.939, 1.076) 0.88015 |

Model I: we did not adjust other covariants;

Model II: we adjusted age, sex, BMI;

Model III: we adjusted age, sex, BMI, ALT, AST, GGT, HDL-c, TG, smoking habits, drinking habits , working habits, hypertension, tumor;

Model IV: we adjusted age (smooth), sex, BMI(Smooth), ALT(Smooth), AST(Smooth); GGT(Smooth), HDL-c(Smooth), TG(Smooth), smoking habits, drinking habits, working habits, hypertension, tumor;

OR, odds ratios; CI: confidence, Ref: reference; HOMA-IR, homeostasis model assessment of insulin resistance; Non-DM: No Diabetes mellitus; DM: Diabetes mellitus;

**Table S4. Association between HOMA-IR and UACR > 30mg/g in diabetes and non-diabetic patients with eGFR＞60 mL/min/1.73m², under different models**

| **Variable** | **Model I (OR,95%CI, P)** | **Model II(OR,95%CI, P)** | **Model III (OR,95%CI, P)** |
| --- | --- | --- | --- |
|  |  | **DM** |  |
| **HOMA-IR** | 0.998 (0.958, 1.040) 0.93558 | 0.988 (0.948, 1.030) 0.56959 | 0.996 (0.955, 1.039) 0.85716 |
|  |  | **Non-DM** |  |
| **HOMA-IR** | 1.054 (1.026, 1.084) 0.00017 | 1.047 (1.018, 1.076) 0.00139 | 1.044 (1.014, 1.074) 0.00346 |

Model I: we did not adjust other covariants;

Model II: we adjusted age, sex, BMI;

Model III: we adjusted age, sex, BMI, ALT, AST, GGT, HDL-c, TG, smoking habits, drinking habits , working habits, hypertension, tumor;

Model IV: we adjusted age (smooth), sex, BMI(Smooth), ALT(Smooth), AST(Smooth); GGT(Smooth), HDL-c(Smooth), TG(Smooth), smoking habits, drinking habits, working habits, hypertension, tumor;

OR, odds ratios; CI: confidence, Ref: reference; HOMA-IR, homeostasis model assessment of insulin resistance; Non-DM: No Diabetes mellitus; DM: Diabetes mellitus;

**Table S5. Association between HOMA-IR and CKD in different models in diabetes with normal and abnormal blood sugar levels**

| **Variable** | **All populations(N=7164)** | **well-controlled group(N=4322)** | **poorly-controlled group(N=2842)** |
| --- | --- | --- | --- |
| **Model I (OR,95%CI, P)** | 0.995 (0.957, 1.034) 0.79012 | 0.954 (0.903, 1.008) 0.09249 | 1.040 (0.984, 1.099) 0.16344 |
| **Model II(OR,95%CI, P)** | 0.983 (0.945, 1.022) 0.38161 | 0.939 (0.888, 0.994) 0.02915 | 1.030 (0.974, 1.090) 0.29369 |
| **Model III (OR,95%CI, P)** | 0.989 (0.950, 1.030) 0.58525 | 0.942 (0.889, 0.998) 0.04141 | 1.039 (0.981, 1.101) 0.19114 |

Model I: we did not adjust other covariants;

Model II: we adjusted age, sex, BMI;

Model III: we adjusted age, sex, BMI, ALT, AST, GGT, HDL-c, TG, smoking habits, drinking habits , working habits, hypertension, tumor;

OR, odds ratios; CI, confidence, Ref, reference; HOMA-IR, homeostasis model assessment of insulin resistance; 2hPG,2 hours postprandial glucose; FBG, fasting blood glucose; well-controlled group (FPG<7.0 mmol/L, 2hPG<11.1 mmol/L, HbA1c<6.5%) and poorly-controlled group (FPG≥7.0 mmol/L, 2hPG≥11.1 mmol/L, HbA1c≥6.5%).

**Table S6 . The results of the two-stage logistic regression model for diabetes with normal and abnormal blood sugar levels**

| Incident diabetes | Model I(OR,95%CI, P ) | |
| --- | --- | --- |
|  | **well-controlled group** | **poorly-controlled group** |
| Fitting model by standard logistic regression | 0.942 (0.889, 0.998) 0.0414 | 1.039 (0.981, 1.101) 0.1911 |
| Fitting model by two-piecewise logistic regression |  |  |
| Inflection point of the HOMA-IR | 2.588 | 1.003 |
| ≤Inflection point | 1.373 (1.202, 1.569) <0.0001 | 3.917 (1.628, 9.425) 0.0023 |
| >Inflection point | 0.720 (0.643, 0.807) <0.0001 | 1.002 (0.941, 1.067) 0.9424 |
| P for log-likelihood ratio test | <0.001 | 0.002 |

We adjusted age, sex, BMI, ALT, AST, GGT, HDL-c, TG, smoking habits, drinking habits, working habits, hypertension, tumor;

OR, odds ratios; CI, confidence, Ref, reference; HOMA-IR, homeostasis model assessment of insulin resistance; 2hPG,2 hours postprandial glucose; FBG, fasting blood glucose; well-controlled group (FPG<7.0 mmol/L, 2hPG<11.1 mmol/L, HbA1c<6.5%) and poorly-controlled group (FPG≥7.0 mmol/L, 2hPG≥11.1 mmol/L, HbA1c≥6.5%).

**Table S7. Effect size of HOMA-IR on the risk of CKD in prespecified and exploratory subgroups**

| Characteristic | Non-DM (OR ,95%CI) | *P* for interaction | DM  (OR ,95%CI) | *P* for interaction |
| --- | --- | --- | --- | --- |
| Gender |  | 0.3263 |  | 0.6518 |
| male | 1.013 (0.965, 1.064) |  | 0.972 (0.913, 1.035) |  |
| female | 1.064 (1.032, 1.098) |  | 1.006 (0.957, 1.058) |  |
| Age(years) |  | 0.4678 |  | 0.4836 |
| <=40 | 0.899 (0.683, 1.184) |  | 1.011 (0.667, 1.532) |  |
| >40, <=50 | 1.059 (0.994, 1.129) |  | 0.954 (0.831, 1.095) |  |
| >50, <=60 | 1.078 (1.034, 1.124) |  | 1.065 (0.997, 1.137) |  |
| >60, <=70 | 1.044 (0.994, 1.098) |  | 0.962 (0.898, 1.030) |  |
| >70 | 0.956 (0.893, 1.024) |  | 0.952 (0.877, 1.033) |  |
| BMI (kg/m^2^) |  | 0.9612 |  | 0.2568 |
| <18.5 | 1.037 (0.879, 1.224) |  | 0.971 (0.692, 1.362) |  |
| >=18.5, <23 | 1.091 (1.043, 1.140) |  | 1.031 (0.948, 1.122) |  |
| >=23 | 1.030 (0.997, 1.064) |  | 0.987 (0.945, 1.032) |  |
| Smoking habits |  | 0.5549 |  | 0.4896 |
| N0ever smoker | 1.016 (0.932, 1.108) |  | 0.974 (0.868, 1.093) |  |
| Sometimes smoker | 1.118 (0.954, 1.310) |  | 1.022 (0.833, 1.255) |  |
| Regular smoker | 1.050 (1.021, 1.080) |  | 0.997 (0.955, 1.040) |  |
| Not record | 1.065 (0.835, 1.358) |  | 0.964 (0.648, 1.435) |  |
| Drinking habits |  | 0.6650 |  | 0.6468 |
| Never drinker | 0.979 (0.879, 1.090) |  | 0.884 (0.750, 1.041) |  |
| Sometimes drinker | 1.050 (0.985, 1.120) |  | 0.995 (0.899, 1.102) |  |
| Regular drinker | 1.057 (1.026, 1.089) |  | 0.999 (0.955, 1.044) |  |
| Not record | 1.096 (0.855, 1.405) |  | 1.335 (0.959, 1.858) |  |
| Exercise status |  | 0.8977 |  | 0.5982 |
| No | 1.028 (0.998, 1.058) |  | 0.979 (0.938, 1.021) |  |
| Yes | 1.035 (0.970, 1.103) |  | 0.978 (0.868, 1.103) |  |
| Not record | 1.847 (1.088, 3.136) |  | 1.005 (0.608, 1.664) |  |
| Tumor |  | 0.2353 |  | 0.9348 |
| Yes | 0.967 (0.830, 1.127) |  | 0.905 (0.742, 1.105) |  |
| No | 1.052 (1.025, 1.080) |  | 1.000 (0.961, 1.040) |  |
| Hypertension |  | 0.1745 |  | 0.2763 |
| No | 1.114 (1.066, 1.164) |  | 1.070 (0.982, 1.167) |  |
| Yes | 1.020 (0.987, 1.053) |  | 0.983 (0.941, 1.026) |  |
| Obesity |  | 0.3625 |  | 0.9427 |
| No | 1.058 (1.028, 1.088) |  | 1.002 (0.959, 1.047) |  |
| Yes | 1.009 (0.946, 1.077) |  | 0.978 (0.900, 1.064) |  |

Note 1: Above model adjusted for age, sex, BMI, ALT, AST, GGT, HDL-c, TG, smoking habits, drinking habits, working habits, hypertension, tumor;

Note 2: In each case, the model is not adjusted for the stratification variable.

OR, odds ratios; CI: confidence, Ref: reference; HOMA-IR, homeostasis model assessment of insulin resistance; Non-DM: No Diabetes mellitus; DM: Diabetes mellitus;


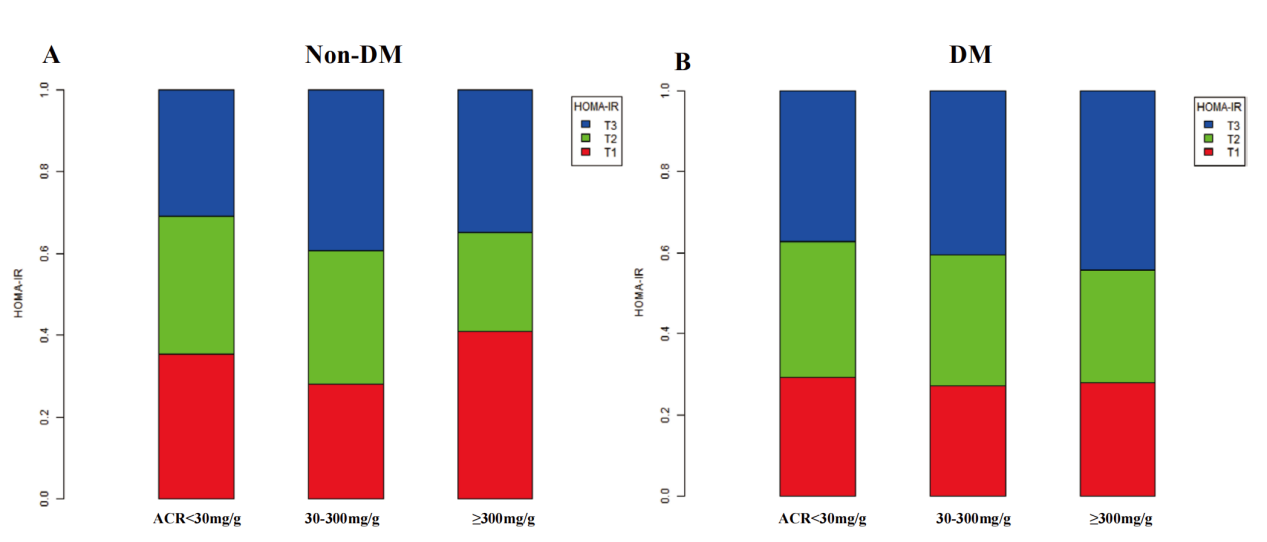


**Figure S1. Distribution of UACR categories by HOMA-IR tertiles in DM and Non-DM cohorts**

Stratified distribution of urinary albumin-to-creatinine ratio (UACR) across HOMA-IR tertiles in diabetic (DM) and non-diabetic (Non-DM) populations. UACR was categorized as normoalbuminuria (<30 mg/g), microalbuminuria (30–300 mg/g), and macroalbuminuria (>300 mg/g). The Non-DM group exhibited a significant increase in microalbuminuria proportion with elevated HOMA-IR tertiles (P < 0.001 for trend), while the DM group showed a blunted rise in macroalbuminuria with no statistical significance. All analyses were adjusted for age, sex, BMI, liver enzymes, lipid profiles, lifestyle factors, hypertension and tumor history. Abbreviations: HOMA-IR, Homeostasis Model Assessment of Insulin Resistance; UACR, urinary albumin-to-creatinine ratio; DM, diabetes mellitus; Non-DM, non-diabetes mellitus; BMI, body mass index.


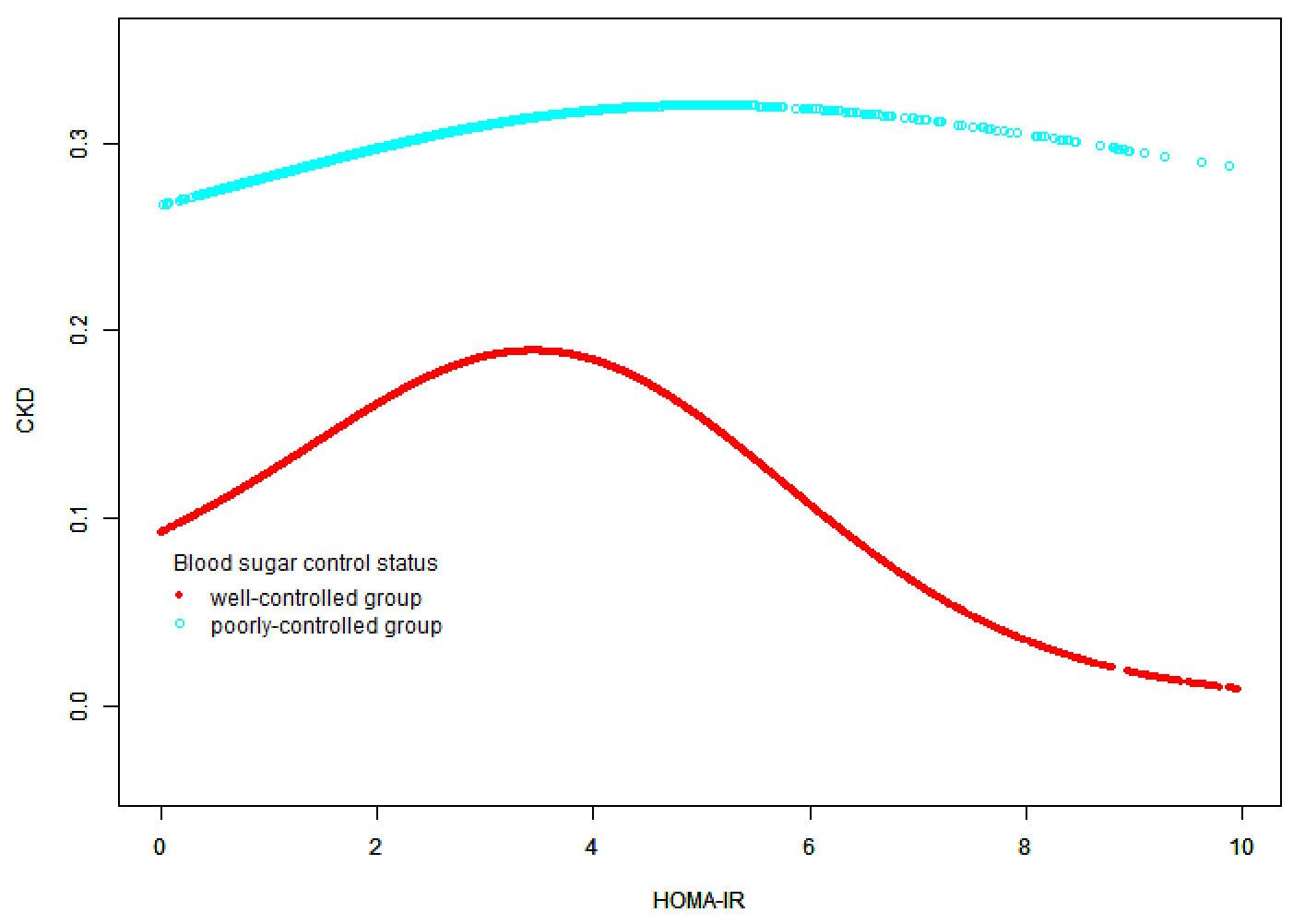


**Figure S2. Relationship between HOMA-IR and CKD in diabetes patients under glycemic control**

The figure displays the relationship between Homeostatic Model Assessment for Insulin Resistance (HOMA-IR, x-axis) and Chronic kidney disease risk (CKD, y-axis) stratified by blood sugar control status. Data points and fitted curves are shown for two groups: well-controlled group (red circles and curve) and poorly-controlled group (cyan circles and curve).
